# The 1.6 Å Crystal Structure of Pyranose Dehydrogenase from *Agaricus meleagris* Rationalizes Substrate Specificity and Reveals a Flavin Intermediate

**DOI:** 10.1371/journal.pone.0053567

**Published:** 2013-01-09

**Authors:** Tien Chye Tan, Oliver Spadiut, Thanyaporn Wongnate, Jeerus Sucharitakul, Iris Krondorfer, Christoph Sygmund, Dietmar Haltrich, Pimchai Chaiyen, Clemens K. Peterbauer, Christina Divne

**Affiliations:** 1 School of Biotechnology, KTH Royal Institute of Technology, Stockholm, Sweden; 2 Department of Medical Biochemistry and Biophysics, Karolinska Institutet, Stockholm, Sweden; 3 Department of Biochemistry and Center of Excellence in Protein Structure and Function, Faculty of Science, Mahidol University, Bangkok, Thailand; 4 Department of Biochemistry, Faculty of Dentistry, Chulalongkorn University, Bangkok, Thailand; 5 Food Biotechnology Laboratory, BOKU University of Natural Resources and Life Sciences, Vienna, Austria; Instituto de Biociencias - Universidade de São Paulo, Brazil

## Abstract

Pyranose dehydrogenases (PDHs) are extracellular flavin-dependent oxidoreductases secreted by litter-decomposing fungi with a role in natural recycling of plant matter. All major monosaccharides in lignocellulose are oxidized by PDH at comparable yields and efficiencies. Oxidation takes place as single-oxidation or sequential double-oxidation reactions of the carbohydrates, resulting in sugar derivatives oxidized primarily at C2, C3 or C2/3 with the concomitant reduction of the flavin. A suitable electron acceptor then reoxidizes the reduced flavin. Whereas oxygen is a poor electron acceptor for PDH, several alternative acceptors, *e.g*., quinone compounds, naturally present during lignocellulose degradation, can be used. We have determined the 1.6-Å crystal structure of PDH from *Agaricus meleagris*. Interestingly, the flavin ring in PDH is modified by a covalent mono- or di-atomic species at the C(4a) position. Under normal conditions, PDH is not oxidized by oxygen; however, the related enzyme pyranose 2-oxidase (P2O) activates oxygen by a mechanism that proceeds *via* a covalent flavin C(4a)-hydroperoxide intermediate. Although the flavin C(4a) adduct is common in monooxygenases, it is unusual for flavoprotein oxidases, and it has been proposed that formation of the intermediate would be unfavorable in these oxidases. Thus, the flavin adduct in PDH not only shows that the adduct can be favorably accommodated in the active site, but also provides important details regarding the structural, spatial and physicochemical requirements for formation of this flavin intermediate in related oxidases. Extensive *in silico* modeling of carbohydrates in the PDH active site allowed us to rationalize the previously reported patterns of substrate specificity and regioselectivity. To evaluate the regioselectivity of D-glucose oxidation, reduction experiments were performed using fluorinated glucose. PDH was rapidly reduced by 3-fluorinated glucose, which has the C2 position accessible for oxidation, whereas 2-fluorinated glucose performed poorly (C3 accessible), indicating that the glucose C2 position is the primary site of attack.

## Introduction

Pyranose dehydrogenase (PDH; *pdh1* gene; pyranose:acceptor oxidoreductase; EC 1.1.99.29; sequence UniProt: Q3L245_9AGAR [1]) from the litter-decomposing fungus *Agaricus meleagris* (synonym *Leucoagaricus meleagris, Agaricus praeclaresquamosus, California fungus*) is an extracellular, monomeric flavin-dependent oxidoreductase, with one flavin adenine dinucleotide (FAD) prosthetic group covalently bound per polypeptide chain [2].

Like several other fungal sugar oxidoreductases, *Am*PDH belongs to the glucose-methanol-choline (GMC) oxidoreductase family [1]; and as the fungal pyranose 2-oxidase from *Trametes multicolor* (*Tm*P2O), *Am*PDH produces aldoketose or diketose derivatives from non-phosphorylated sugars [2]. PDH transcription is up-regulated during limiting oxygen supply, suggesting that PDH may substitute for oxidases under oxygen-deprived conditions [1]. PDHs can be differentiated from P2Os based on: *^i)^* PDH is a glycosylated, extracellularly secreted enzyme, whereas P2O is non-glycosylated and located in the hyphal periplasmic space; *^ii)^* PDH is rather inert towards oxygen, while P2O is a typical flavoprotein oxidase; and *^iii)^* PDH oxidizes D-glucose at both the C2 and C3 position, whereas P2O is strictly regioselective for the glucosyl C2 position [3]–[6].

Interestingly, the two enzymes PDH and P2O, which catalyze closely related reactions, appear to be mutually exclusive. Of all fungal strains screened for P2O and PDH activity, they were found to express either PDH or P2O, but never both [7]. Moreover, the enzyme requirement of a particular fungus appears to be coupled to the macroscopic substrate. Volc and co-workers showed that P2O is expressed mainly by wood-decaying white-rot fungi (e.g., *Phanerochaete*, *Trametes etc.*), while PDH expression is limited to the *Agaricales* [7], which are typical litter-degrading fungi that live in forest and grassland soil and are the primary decomposers of residual plant material (leaves, needles, twigs, bark, grass) in the uppermost soil layer. It should be noted that P2O-encoding genes were also found and experimentally confirmed in some members of the genus *Aspergillus*, which does not degrade lignocellulose, notably in species where a glucose 1-oxidase (GOX) is absent [8].

At present, the biologically relevant function of PDH is not clear. One possible role of PDH would be the reduction of quinone compounds or reactive radical species generated during lignin depolymerization, possibly to prevent re-polymerization or to prevent exposure of the cell to toxic quinones. A similar role has been suggested for basidiomyceteous P2O and cellobiose dehydrogenase (CDH) for wood-degrading fungi [9]–[11]. Although *A. meleagris* feeds mainly on lignocellulose-rich forest litter like straw or bark, and compact wood is usually not degraded [12], one may hypothesize that PDH performs a similar function in litter-decomposing fungi to that of CDH. The ability of PDH to oxidize at comparable activities a wide range of carbohydrates present in wood, *e.g*., D-xylose, L-arabinose D-glucose, D-galactose, cellobiose, and others [13], [14], together with its up-regulation once easily metabolizable carbohydrates are depleted from the medium, strongly support a role in the degradation process.

**Figure 1:**
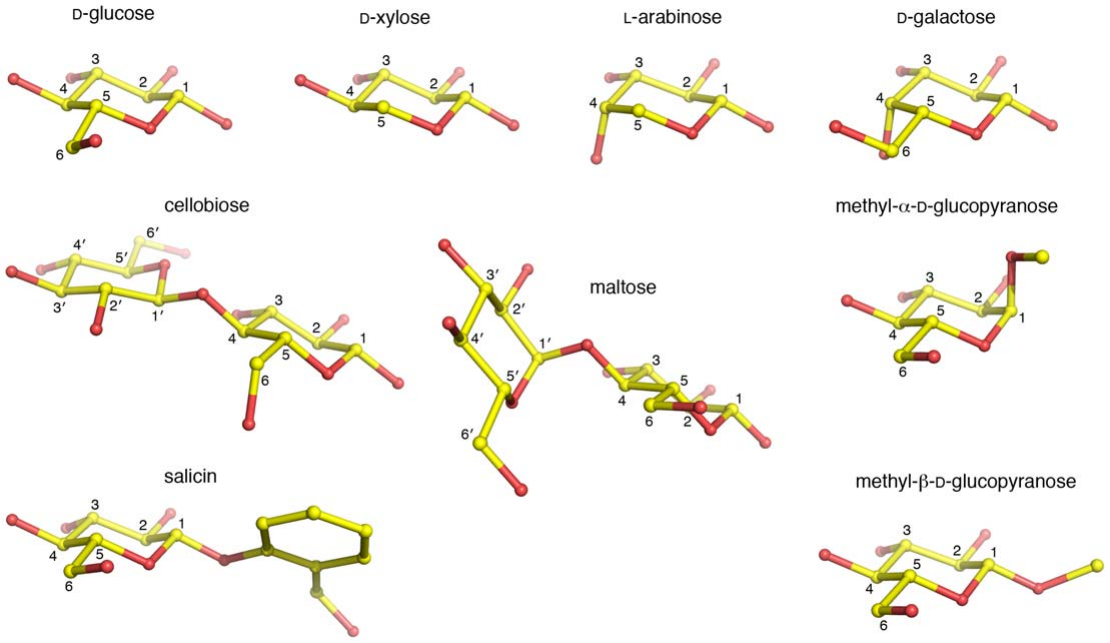
Electron-donor substrates modeled in the active site of *Am*PDH. Carbons are colored yellow and numbered. Apostrophes indicate carbons on the non-reducing end sugar. Hydrogen atoms have been omitted for the purpose of clarity.

The fungus *A. meleagris* carries three genes that putatively encode pyranose dehydrogenases, *pdh1*, *pdh2* and *pdh3*. The latter two genes are transcribed on a much lower level than *pdh1* [1], and the PDH protein isolated and characterized from cultures of *A. meleagris* in previous studies [2], [13], [15] also corresponds exclusively to *pdh1*. The product of *pdh1*, *Am*PDH, is a glycoprotein with a molecular mass of 66.5 kDa and an estimated 7% glycan content [2]. The FAD cofactor displays typical absorption peaks at 371 nm and 464 nm for the oxidized enzyme, whereas the reduced enzyme lacks the 464 nm peak [2]. The optimal pH stability range is broad (pH 4–10), and the temperature optimum is 63°C under standard assay conditions [16]. The enzyme has a broad electron-donor substrate specificity (Fig. 1), including a range of mono- and oligosaccharides where D-glucose, D-galactose, L-arabinose, and D-xylose are all highly competent electron donors when ferricenium is used as an electron acceptor. Suitable electron acceptors are either complexed metal ions or substituted quinones. Of the electron acceptors tested, ferricenium performs best, followed by 3,5-di-tert-butyl-benzoquinone and 2,6-dichloroindophenol [2].

Regioselectivity in sugar oxidation strictly differs depending on the substrate used. An extensive study by Sedmera and co-workers [13] has shown that PDH is able to oxidize sugar substrates through monooxidation reactions at the C1, C2, C3 positions, or double-oxidations at C1/3, C2/3, or C3/4. Whereas D-glucose is double-oxidized at C2 and C3 to yield 2,3-didehydro-D-glucose, D-galactose (the C4 epimer of glucose) is oxidized exclusively at C2 to give 2-dehydro-D-galactose. Similar to D-galactose, L-arabinose is oxidized at C2 yielding 2-dehydro-L-arabinose. Cellobiose, maltotriose, D-xylose and maltose are all double-oxidized at C2 and C3. The double-oxidation reactions of cellobiose and maltose resulted in the novel compounds 2,3′-didehydrocellobiose and 2,3′-didehydromaltose, respectively. Lactose is oxidized at the reducing-end C1 or C2 position to give lactobionolactone and 2-dehydrolactose [17]. The monosaccharides D-ribose, D-allose (C3 epimer of glucose), D-gulose (C3 epimer of galactose), and D-talose (C2 epimer of galactose) are oxidized exclusively at C1 to the corresponding aldonic acids. Interestingly, *Am*PDH has been shown to perform double-oxidation of a number of aromatic glycosides, of which β-D-glucopyranosides and a β-D-xylopyranoside are converted to novel sugar compounds corresponding to the 3,4-didehydro-β-D-aldopyranoside forms [15]. The reactions carried out by PDHs can be summarized as:

1. pyranose+acceptor = 2-dehydropyranose (or 1-dehydropyranose or 3-dehydropyranose or 2,3-didehydropyranose)+reduced acceptor
2. pyranoside+acceptor = 3-dehydropyranoside (or 3,4-didehydropyranoside)+reduced acceptor

Based on its primary structure, PDH belongs to the same flavoenzyme family as the oxygen-activating enzymes *Tm*P2O, *Aspergillus niger* glucose 1-oxidase (*An*GOX), which oxidizes β-D-glucose to D-gluconolactone [18], and *Arthrobacter globiformis* choline oxidase (*Ag*CHO), which oxidizes choline to glycine betaine [19]. Unlike *Am*PDH, the oxidases *Tm*P2O, *Ag*CHO and *An*GOX use molecular oxygen as electron acceptor to generate hydrogen peroxide. Given the structural, functional and mechanistic kinship between these enzymes, the crystal structure of *Am*PDH also gives us the opportunity to identify structural features that may help rationalize the difference in oxygen reactivity, *i.e*., structural determinants underlying the designation of a GMC enzyme as an oxidase or a dehydrogenase.

Here we report the crystal structure of *Am*PDH (the translated product of *pdh1*), refined at 1.6 Å resolution, featuring a C(4a)-modified flavin ring. Based on the PDH structure, we rationalize the observed pattern of sugar substrate preference and regioselectivity, and discuss the relevance of the flavin adduct species with respect to the biological function of PDH. The surrounding environment of the C(4a)-flavin adduct in PDH is compared with those of related flavoprotein oxidases and monooxygenases.

## Results

### Activity on 2- and 3-Fluorinated Glucose

**Figure 2:**
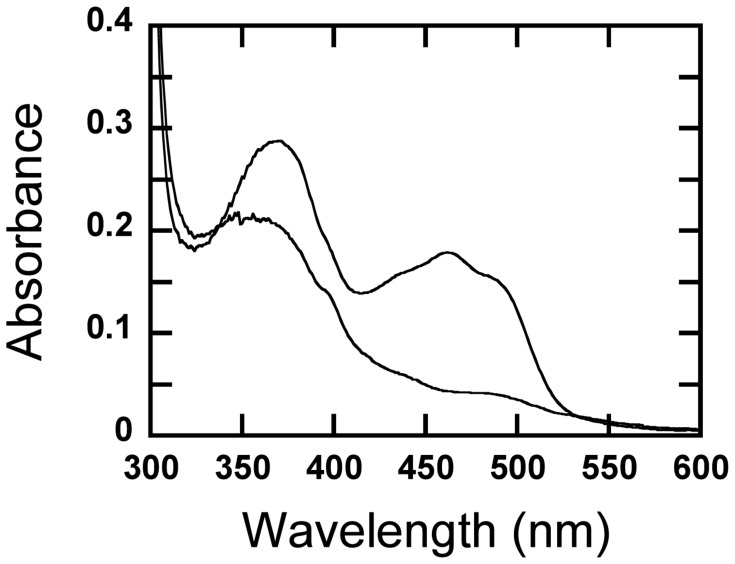
Reduction of oxidized *Am*PDH by 3-fluoro-3-deoxy-D-glucose. The upper spectrum represents the absorption spectrum of the oxidized PDH (20 µM), and the lower spectrum shows the reduced enzyme after incubation with 20 µM 3FG for 30 sec at 25°C. The reduction by 3FG (presumably by a hydride transfer from C2-H) is very rapid and cannot be followed kinetically by manual mixing in an anaerobic cuvette.

**Figure 3:**
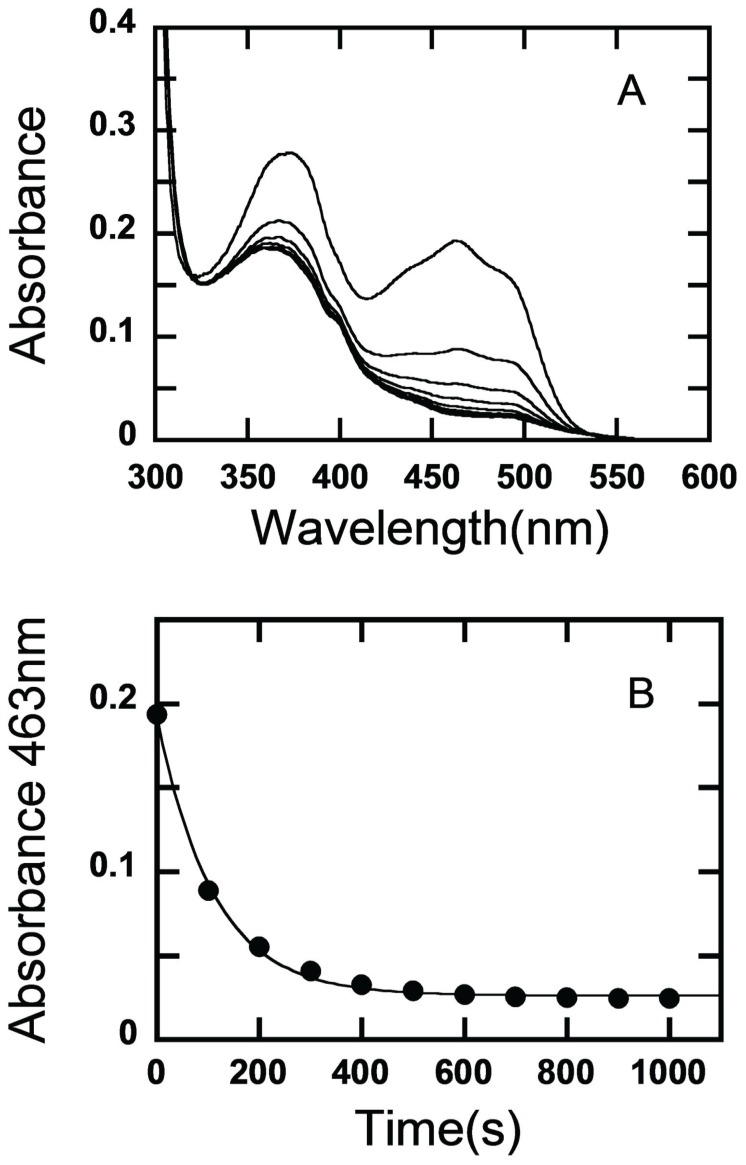
Reduction of oxidized AmPDH by 2-fluoro-2-deoxy-D-glucose. (A) Reduction spectra of PDH (20 µM) reduced by 2FG (20 µM) at 25°C. The top spectrum represents the absorption spectrum of the oxidized PDH. Spectra were recorded at intervals during the reaction. The upper spectrum represents the start of the reaction, and the lowest spectrum the end (*t* = 1000 sec) showing PDH in its fully reduced state. (B) Reduction kinetics of PDH with 2FG was monitored at 463 nm. The reaction kinetics corresponds to a *k*_obs_ value of 0.0091 sec^−1^.

When mixing 3-fluoro-3-deoxy-D-glucose (3FG) and *Am*PDH, the reaction mixture monitored at 30 s after mixing was colorless (Fig. 2) showing that *Am*PDH was rapidly reduced, presumably by a hydride moiety at glucose C2. The rate of reduction of PDH by 2-fluoro-2-deoxy-D-glucose (2FG) was significantly slower because the reduction kinetics could be monitored over a period of 1000 s (Fig. 3*a*). Fig. 3*b* shows the reduction kinetics of PDH by 2FG monitored at 463 nm, which is consistent with an observable rate constant (*k*_obs_) of 0.0091 s^-1^.

### Oxygen Activity

**Figure 4:**
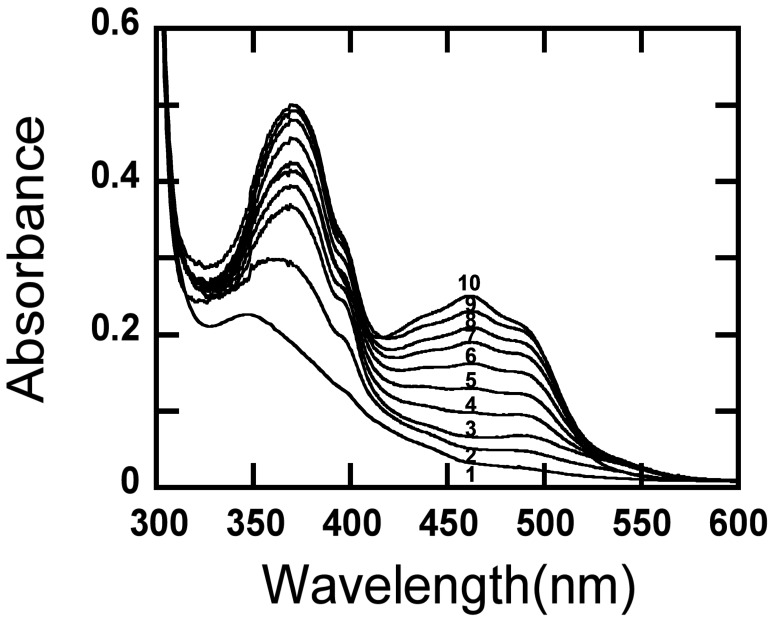
Absorption spectra of reduced *Am*PDH reacting with molecular oxygen. Oxidation of PDH by oxygen at air-saturation as a function of time at 25°C. Line 1: reduced PDH at start of the reaction which lacks the flavin absorption shoulder around 490 nm; line 5, the spectrum of partially oxidized PDH (∼40% oxidation); line 10, the absorption spectrum of partially oxidized PDH after the reaction proceeded for 15 h.

The absorption spectra of *Am*PDH undergoing oxidation by oxygen at air-saturation over a time period of 15 h are shown in Fig. 4. The reaction proceeded slowly, and the enzyme was still only partially oxidized after 15 h. After 15 h, enzyme denaturation occurred as judged by increased turbidity of the solution. The spectra in Fig. 4 indicate absorption peaks ∼370 nm and resolving shoulders around 395 and 495 nm, which are characteristic of a flavin anionic semiquinone.

### Overall Structure

**Figure 5:**
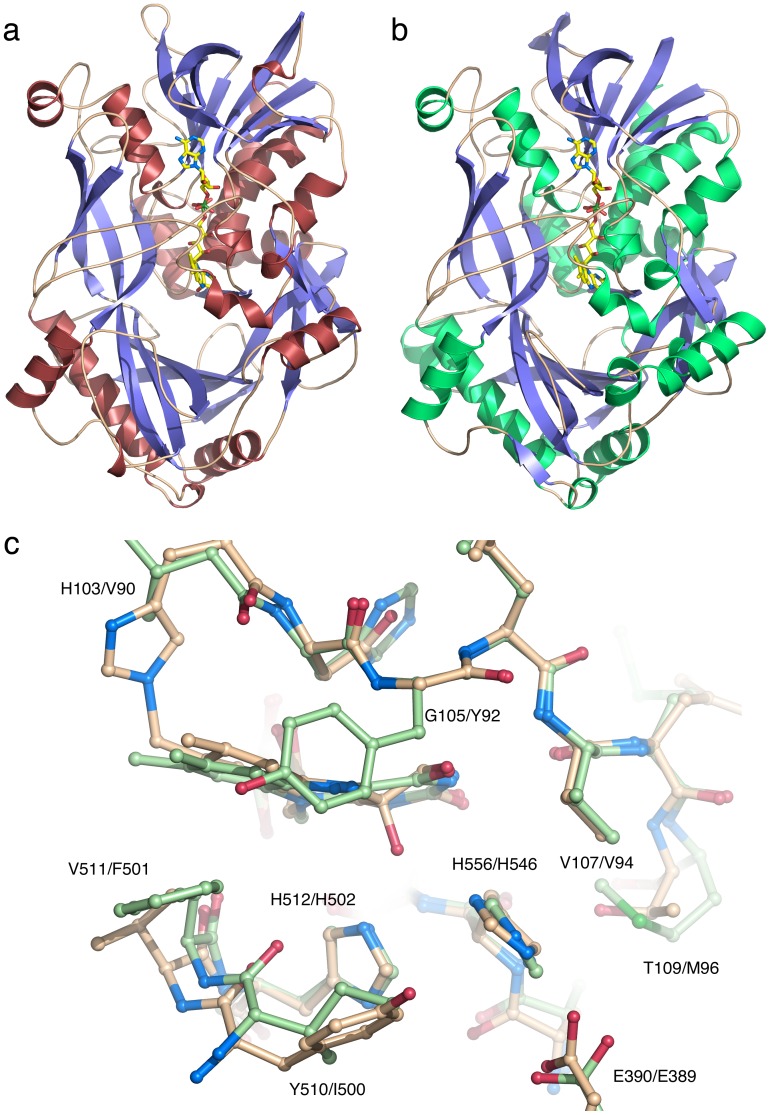
Overall structure of *Am*PDH and similarity to AAO. Ribbon drawing of *Am*PDH (a) and aryl-alcohol oxidase (b), showing α-helices as spirals and β-strands as arrows. The covalently bound flavin cofactor is depicted as a stick model with carbon atoms in yellow. An overlay picture of the active site in *Am*PDH (beige carbon atoms) and AAO (green carbon atoms) is shown in (c).

The overall structure of *Am*PDH features the classical p-hydroxybenzoate hydroxylase (PHBH)-like fold of members of the GMC oxidoreductase family with two intimately associated domains, an ADP-binding Rossmann domain, and a substrate-binding domain (Fig. 5a). Data collection and model refinement statistics are given in Table 1. The GMC member most structurally similar to PDH returned by the Dali server (DaliLite v.3; http://www.ebi.ac.uk/Tools/dalilite/ [20]) is aryl-alcohol oxidase (AAO; PDB code 3FIM [21]) with a root-mean-square distance (r.m.s.d) of 1.6 Å for 544 of 575 aligned Cα atom pairs, and a sequence identity of 38% (Fig. 5b, c). Of the GMC members using carbohydrates as electron-donor substrates, optimized structural alignments with PDH were obtained using the *LSQ_IMPROVE* option in the program *O* [22], giving r.m.s.d. values of 1.5 Å for GOX (PDB code 1CF3 [23], 461 Cα pairs); 1.6 Å for CDH (PDB code 1NAA [24], 387 Cα pairs), and 2.0 Å for P2O (PDB code 3PL8 [6], 313 Cα pairs).

**Table 1:** Data collection and crystallographic refinement statistics for *Am*PDH.

Data collection^a^	
Cell constants a, b, c (Å)	51.94, 74.64, 139.29
Space group/moleculesper asymmetric unit	*P*2_1_2_1_2_1_/1
Beamline, λ (Å)	*I*911–5, 0.90772
Resolution range, nominal (Å)	48.7–1.60 (1.70–1.60)
Unique reflections	72,035 (11,811)
Multiplicity	7.3 (7.3)
Completeness (%)	99.7 (99.8)
<*I*/σ*I*>	16.4 (2.6)
*R_sym_* b (%)	7.5 (84.1)
*CC*(1/2)^c^	99.9 (80.1)
**Crystallographic refinement**	
Resolution range (Å)	50–1.60 (1.686–1.600)
Completeness, all % (outer bin)	99.7 (99.8)
*R_factor_* d/work reflns, all	0.171/69,857
*R_free_*/free reflns, all	0.201/2,177
Non-hydrogen atoms	5,017
Mean *B* (Å^2^) protein all/mc/sc	15.6/14.5/16.7
Mean *B* (Å^2^) solvent/N^o^. mol.	29.9/541
Rmsd bond lengths (Å), angles (°)	0.016, 1.853
Ramachandran: favored/allowed (%)^e^	97.2/100

a The outer shell statistics of the reflections are given in parentheses. Shells were selected as defined in *XDS* [54] by the user.

b *R_sym_* = [Σ*_hkl_* Σ*_i_* |*I–<I>*|/Σ*_hkl_* Σ*_i_* |*I*| ] x 100%.

c CC(1/2) = Percentage of correlation between intensities from random half-datasets. Values given represent correlations significant at the 0.1% level [66].

d *R_factor_* = Σ*_hkl_* | |F_o_|–|F_c_| |/Σ*_hkl_* |F_o_|.

e As determined by *MolProbity* [62].

### Active-Site Structure

**Figure 6:**
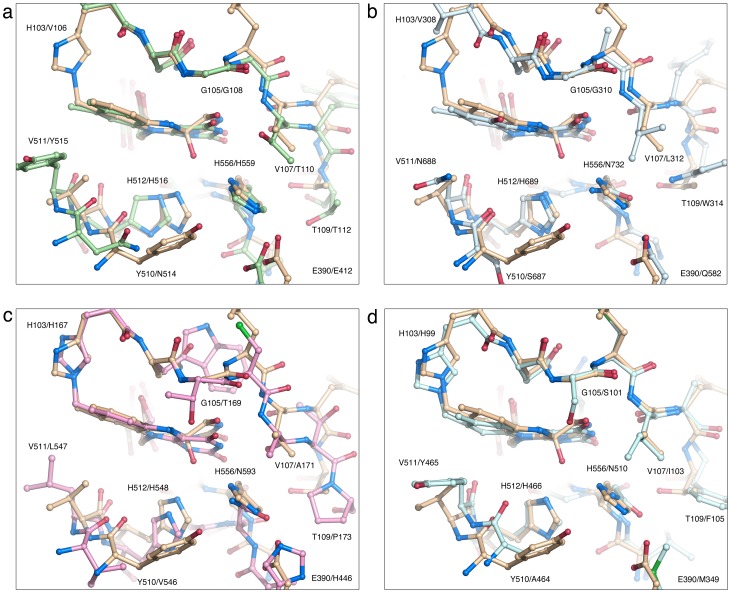
Comparison of *Am*PDH with GOX, CDH, P2O and CHO. Structural superpositioning of the *Am*PDH active site (beige carbon atoms) with those of (a) GOX (PDB code 1CF3 [23]), green carbons; (b) CDH flavoprotein domain (PDB code 1KDG [30]), light blue carbons; (c) P2O (PDB 1TT0 [46]), pink carbons; and (d) CHO (PDB 3LJP [67]), pale cyan carbons. For each equivalent pair, that of AmPDH is given first.

**Figure 7:**
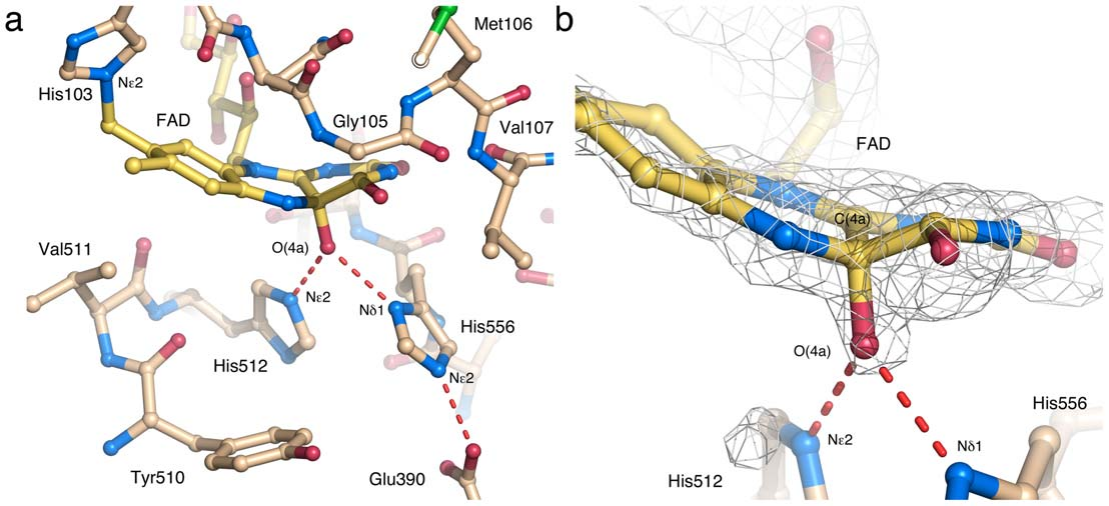
Structural evidence for a flavin C(4a)-adduct. (a) The AmPDH active site showing the relevant amino acids including the catalytic His-His pair. The FAD molecule is covalently linked through the C8M atom to His103 Nε2, and features a modification at the C(4a) locus. The adduct-stabilizing imidazole nitrogens of His512 and His556 are pictured with dashed lines indicating hydrogen bonds. (b) Zoom in at the monoatomic flavin-adduct species, presumably a covalently bound oxygen atom, overlaid by a 5000 K simulated annealing omit electron density map calculated using *PHENIX* [63], contoured at the 3σ level.

Of closely related GMC members that use sugars as electron-donor substrates, three-dimensional structural information exists for two oxidases (P2O and GOX) and two dehydrogenases (PDH and CDH). A structural overlay of PDH with GOX, P2O and CDH (Fig. 6*a, b, c*) highlights the similarities and differences between the enzyme active sites. For the purpose of discussing oxygen reactivity, CHO has been included (Fig. 6*d*). CHO does not oxidize sugar substrates, but its structure has been determined with a covalent C(4a) flavin modification that is probably an artifact introduced during data collection. Although the overall similarity of the active sites is high, some differences are noted (Fig. 6, Table 2): *i)* PDH and GOX contain a His-His catalytic pair, whereas P2O, CDH and CHO use a His-Asn constellation; *ii)* PDH, P2O and CHO are flavinylated (8α-(*N*3)–histidyl–FAD), while GOX and CDH carry non-covalently bound FAD; and *iii)* PDH, GOX and CDH lack a *si*-face N(5) partner, *i.e.*, the side chain close to N(5) that forms hydrogen bonds with the N(5)/O(4) locus, but P2O and CHO contain a Thr and Ser, respectively. Unexpectedly for a flavoprotein dehydrogenase, the flavin ring in *Am*PDH is modified at C(4a) by an unidentified atomic species (*Fig. 7*). The covalently bound atom (probably an oxygen species) is tightly coordinated by the two active site histidines (512 and 556).

**Table 2:** Comparison of related GMC dehydrogenases and oxidases.

Enzyme^a^ (PDB code)	Physiol.O_2_ activity	Catalytic pair	N-N distance catalytic pair (Å)	Physiol. flavinC(4a) adduct	Flavin attachment	*si*-face N(5) partner
*Am*PDH (this work)	No	His512 Nε2, His556 Nδ1	3.5	No, radiationartifact	8α-(*N*3)–histidyl–FAD	Gly105
*Pc*CDH (1KDG [30])	No	His689 Nε2, Asn732Nδ2	3.3	No	Non-covalent	Gly310
*Tm*P2O (1TT0 [46])	Yes	His548 Nε2, Asn593 Nδ2	4.2	Yes	8α-(*N*3)–histidyl–FAD	Thr169
*Ag*CHO (3LJP [67];2JBV [25])	Yes	His466 Nε2, Asn510 Nδ2	4.0	No, radiationartifact	8α-(*N*3)–histidyl–FAD	Ser101^b^
*An*GOX (1CF3 [23])	Yes	His516 Nε2, His559 Nδ1	4.8	No	Non-covalent	Gly108

a *Am*PDH, *A. meleagris* pyranose dehydrogenase; *Pc*CDH, *P. chrysosporium* cellobiose dehydrogenase; *Tm*P2O, *T. multicolor* pyranose 2-oxidase; *An*GOX, *A. niger* glucose 1-oxidase; *Ag*CHO; *A. globiformis* choline oxidase.

b The *Ag*CHO mutant S101A shows increased efficiency in the oxidative half-reaction [68], stressing that the function of this side chain is different in *Ag*CHO compared with the sugar-oxidizing enzymes.

### Comparison of Flavin C(4a)-Adduct Geometry in Existing Crystal Structures

The C(4a)-modified flavin in *Am*PDH shows an *sp* ^3^-hybridized C(4a) atom, and a relatively subtle conformational change compared with a planar isoalloxazine ring (Fig. 7). A detailed analysis of the flavin geometry is provided in Table S1, where the *Am*PDH adduct is compared with the flavin C(4a) adduct of *Ag*CHO (PDB code 2JBV [25]), and the available crystal structures of artificial synthetic isoalloxazine oxygen-nucleophile adducts, O-adducts [26]. Specifically, the structures of two flavin C(4a)-OH adducts (1AOH, CCDC code 784805; 1BOH, CCDC code 784806) allow a detailed characterization of the adduct geometry. The two flavin OH-adducts, 1AOH and 1BOH, correspond to isoalloxazine and alloxazine (lacking the C7 and C8 methyl groups), respectively. To stabilize the flavin adducts, the (iso)alloxazine rings were modified at N(5) to yield the 5-alkyl flavin analogs prior to adduct formation [26]. As a result, these artificial C(4a) O-adducts are formed at the *si*-face of the flavin rather than the *re*-face observed in the crystal structures of the flavoprotein oxidases, but we do not expect this difference to be of any major importance for a comparison of O-adduct geometry. The substituted C(4a) atoms in 1AOH and 1BOH display *sp* ^3^ character with an average value for the bond angles at C(4a) close to 109.5° (expected for a tetrahedral carbon), resulting in a flavin-ring distortion with a small but distinct deviation from planarity [26].

For the purpose of analyzing the differences in flavin ring conformation for different O-adducts, we find the dihedral angle N(10)–C(10)–C(4a)–C(4) to be useful in that it provides a measure of the distortion of the pyriminoid ring (the ring that is most affected by O-substitution in the 1BOH, *Am*PDH and CHOx adducts). This torsion angle relates the N(10) atom of the pyrazinoid ring to the C(4) atom of the pyriminoid ring, and a value of ±180° would mean that these atoms are in one plane, and *trans* to each other. The values reported for the two *si*-face O-adducts 1AOH and 1BOH are 174.5° and 139.7° [26]. For *re*-face flavin O-adducts (as those in *Am*PDH and CHO) these values would translate into the counterclockwise rotations of –174.5° and –139.7°.

**Figure 8:**
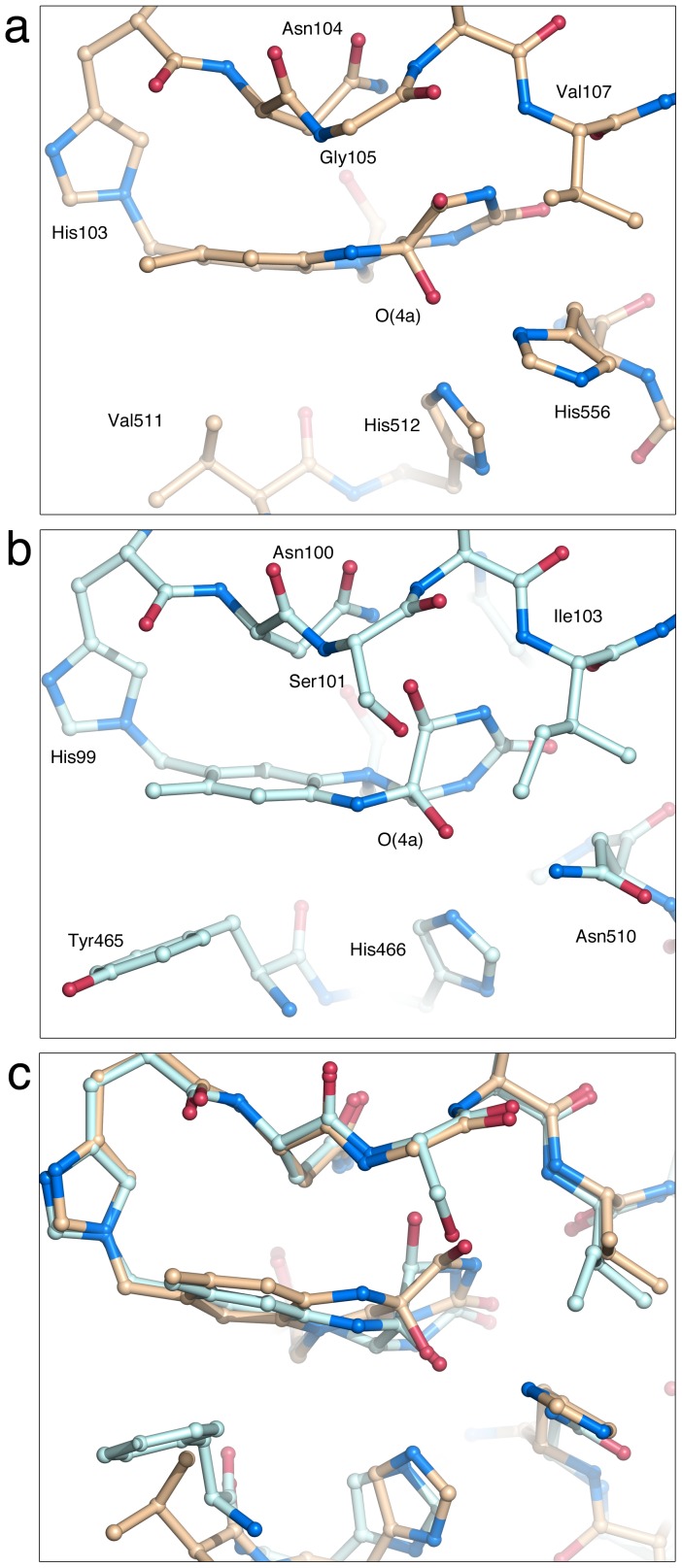
Comparison of the flavin C(4a) adduct in *Am*PDH and CHO. The active site in (a) *Am*PDH (this work; beige carbons) and (b) *Ag*CHO (rerefined model of 2JBV [38], unpublished; light blue carbons) highlighting the modified FAD cofactor. (c) Overlay of the images in (a) and (b). The distortion of the flavin ring accompanying adduct formation is more subtle in *Am*PDH compared with *Ag*CHO.

For *Ag*CHO, Orville and co-workers reported the crystal structure of a trapped flavin C(4a) adduct, as well as density functional theory (DFT) calculations. Based on the DFT calculations, values for the N(10)–C(10)–C(4a)–C(4) angle for the C(4a)–OH, C(4a)–OOH, FAD semiquinone, and FADH^-^ fall in the range –130° to –156°, whereas the value of this dihedral angle in the *Ag*CHO crystal structure (PDB code 2JBV) is 86°. The observed value of –152° for the N(10)–C(10)–C(4a)–C(4) dihedral angle in the *Am*PDH adduct (Table S1) agrees reasonably well with those generated by DFT calculations [25] and with the synthetic flavin O-adducts [26], but is distinct from the modified flavin in *Ag*CHO. Thus, the adduct geometry in the *Ag*CHO structure differs about 40° from the DFT values for C(4a)–OH/C(4a)–OOH, and 50–90° from those of the synthetic adducts and *Am*PDH. Indeed, this torsion angle emphasizes the fundamentally different conformation of the pyriminoid ring in *Ag*CHO compared with that in *Am*PDH (Fig. 8), and synthetic flavin O-adducts.

### Modeling of Monosaccharide Electron-Donor Substrates in the *Am*PDH Active Site

Electron-donor substrates (saccharides) were docked in the *Am*PDH active site to rationalize the observed patterns of substrate specificity and regioselectivity [2], [13]. Only substrates for which the activity exceeds 50% of the activity for D-glucose, and with experimentally determined site(s) of oxidation, were considered (according to compiled data [13]; Fig. 1). A summary of possible protein-sugar hydrogen bonds for monosaccharide and disaccharide substrates is given in Table S2 and Table S3, respectively. Based on our structural modeling, *Am*PDH should be able to oxidize C1, C2, C3 and C4 of D-glucose and D-xylose [27] (the only difference being that xylose lacks the C6–O6 group) without any obvious problems (Fig. S1 and S2). All oxidation-binding modes generate similar sets of possible hydrogen bonds, which explain the more promiscuous oxidation activity of *Am*PDH compared with *Tm*P2O for these monosaccharides. Single oxidations at C1 and C4 have not been observed experimentally, but should be possible from a purely structural point of view (Fig. S1 *a,d* and S2*a,d*). For all sugar-binding modes, the principal interactions are provided by the catalytic histidine pair (His512/His556), the backbone carbonyl oxygen of Tyr510, and the side chain of Gln392. The C6 hydroxyl group does not form any interactions in either of the modeled binding modes. Since the two binding modes, the C2-oxdiation and C3-oxidation modes, generate similar sets of interactions (Fig. S1 *b,c*), their difference in kinetics (see above) is difficult to rationalize at the structural level.

Unlike D-glucose and D-xylose, oxidations of L-arabinose (Fig. S3) and D-galactose, the C4 epimer of glucose, (Fig. S4) are highly regioselective reactions, targeting only the C2 position and strongly discriminating against C1, C3 and C4. Thus, for D-galactose, only 2-dehydro-D-galactose is produced [28], and for L-arabinose, which is identical to D-galactose except for the absence of an exocyclic C6–O6 group, only 2-dehydro-L-arabinose [13]. The reason for the observed product outcome is easily rationalized at the structural level using modeling.

When L-arabinose and D-galactose are oriented for oxidation at C2, the axial O4 is easily accommodated in the active site, whereas in all other orientations, *i.e.,* oxidation at C1, C3 and C4, the axial O4 group would experience steric hindrance and generate clashes (qualititatively defined as a distance less than the sum of the van der Waals radii for any two non-bonded atoms, and not participating in short-strong hydrogen bonds or low-barrier hydrogen bonds). In orientation for C1 (Fig. S3*a* and S4*a*) or C3 (Fig. S3*c* and S4*c*) clashes are observed with the aromatic ring of Tyr510 in subsite C, and binding for oxidation at C4 results in equally unfavorable clashes, but with the flavin ring (Fig. S3*d* and S4*d*). In contrast, the C2-oxidation mode enables four possible protein-sugar hydrogen bonds (Fig. S3 *b* and S4*b*), and no unfavorable interactions, yielding L-*erythro*-pentos-2-ulose [13] and D-lyxo-hexos-2-ulose [2], [28] from L-arabinose and D-galactose, respectively.

Oxidations of PDH by the glucopyranosides methyl-α-D-glucopyranose [29] (methyl-α-D-Glc*p*; Fig. S5) and methyl-β-D-glucopyranose (methyl-β-D-Glc*p*; Fig. S6) are also highly regioselective, favoring oxidation at C3, all of which is substantiated by our modeling (Fig. S5 *c* and S6*c*). For oxidation at other positions of methyl-α-D-Glc*p* (Fig. S5 *a, b, d*) the axially configured methoxy group will generate clashes with either the flavin ring or the tyrosine 510 ring. In principle, the same applies to methyl-β-D-Glc*p*: in the C1-oxidation mode, the methoxy group experiences steric problems with the flavin ring (Fig. S6 *a*), and in the C2-oxidation mode it clashes with Val511 (Fig. S6 *b*). Although C4-oxidation has, to our knowledge, not been shown for methyl-β-D-Glc*p*, this nonetheless appears possible from a structural point of view (Fig. S6 *d*).

### Modeling of Disaccharide and Glucopyranoside Electron-donor Substrates in the *Am*PDH Active Site

For binding of disaccharides or glucopyranosides substituted with large aglycons, we use the subsite-naming convention previously defined for CDH [24], [30]. According to this convention, the innermost site where oxidation takes place is referred to as subsite C (indicating that this is the catalytic site), and the second binding site is named B1 (“B” for binding). Longer oligomers would then involve additional subsites, B2, B3 *etc*. The disaccharides cellobiose and maltose consist of two linked glucosyl units, but with different glycosidic linkages: a β(1→4) glycosidic bond in cellobiose, and an α(1→4) linkage in maltose. In both cases, PDH catalyzes single oxidations at C1 and C2 of the reducing-end glucosyl unit, *i.e.,* 1- and 2-oxidation; and at C3 of the non-reducing glucosyl terminus, *i.e.,* 3′-oxidation [13] (positions denoted by an apostrophe refer to positions in the non-reducing end sugar). In addition to the single oxidations, PDH also catalyzes the sequential double-oxidation reactions 1,3′ (C1 oxidation of reducing-end glucosyl and C3 oxidation of non-reducing end glucosyl) or 2,3′ (C2 oxidation of reducing-end glucosyl and C3 oxidation of non-reducing end glucosyl), to yield the 1,3′ or 2,3′-double-oxidation products [2]. The products from 1,3′-double-oxidation reactions are 3′-dehydrocellobionic acid and 3′-dehydromaltobionic acid, and from 2,3′-double-oxidation, products 2,3′-didehydrocellobiose and 2,3′-didehydromaltose are obtained.

The product outcome is straightforward to rationalize based solely on structural considerations (cellobiose, Fig. S7; maltose, Fig. S8): favorable interactions are only possible when the C-site sugar unit is oriented for oxidation at C1 or C2 (Fig. S7*a, b* and S8*a, b*). The 3- (Fig. S7*c* and S8*c*) and 2′-oxidations (Fig. S7*d* and S8*d*) are discriminated against due to severe steric clashes incompatible with substrate binding: in the case of 3-oxidation, extreme clashes are observed between the non-reducing end sugar and the Tyr510-Val511 backbone (Fig. S7*c* and S8*c*); and for 2′-oxidation, clashes are formed between the reducing end glucose and Tyr510 (Fig. S7*d* and S8*d*).

In the case of the 3′-oxidation mode for cellobiose (C3 of the non-reducing end glucosyl positioned for oxidation in subsite C; Fig. S7*e*), we observe no possible interactions for the reducing-end glucosyl unit in site B1, however, a minor pivotal rotation of the subsite B1 glucosyl together with a concomitant rotamer shift of the Ser64 side chain generates a possible additional hydrogen bond O1–Ser64 Oγ. In the case of maltose, the 3′-oxidation activity is more difficult to rationalize at the structural level since most positions appear to result in clashes either with the Gly105 or Ser64 backbone in subsite B1 (Fig. S8*e*). The clashes are however not severe, and small adjustments of the protein backbone regions could easily relieve unfavorable energies.

Although the 4′-oxidations of cellobiose (Fig. S7*f*) or maltose (Fig. S8*f*) have not been reported for *Am*PDH, these reaction may be possible from a purely structural point of view. Analysis of the maltose 4′-oxidation mode (Fig. S8*f*) indicates that this mode may be somewhat difficult to accommodate with respect to the Tyr510 ring, which forms a “floor” below the sugar ring positioned for oxidation in subsite C. The steric hindrance does not seem severe, but considering the potentially important role of Tyr510 to ensure optimal induced fit, even minor spatial problems in this region are likely to be incompatible with productive substrate-binding modes. Thus, for the disaccharides cellobiose and maltose, the 1 and 2 positions in the reducing-end glucosyl unit are indeed the only sites easily accessible for oxidation. Moreover, placing the non-reducing end glucosyl unit of cellobiose, or maltose, in subsite C unambiguously shows that only C3 and C4 are possible, *i.e.,* 3′- and 4′-oxidation.

Salicin is a glucopyranoside substituted at O1 with an *ortho*-benzylic alcohol ring present in willow bark and known for its properties as a natural analgesic. In the C1- and C2-oxidation modes (Fig. S9*a, b*), the benzylic ring of salicin forms severe clashes with either the flavin ring or the Tyr510 backbone. Favorable interactions are formed only when the non-reducing end glucose unit is positioned in site C for 3- or 4-oxidation (Fig. S9*c, d*).

## Discussion

### Electron-donor Substrate Preference

The similarity in electron-donor substrate and active-site structure of *Am*PDH, GOX and P2O offers a framework to study substrate selectivity and regioselectivity. The active site of *Am*PDH is more similar to that of *An*GOX than to *Tm*P2O, however, regioselectivity differs for the sugar substrate: *An*GOX oxidizes exclusively at the glucosyl C1 position, whereas *Am*PDH can perform single oxidations at the endocyclic glucosyl-ring positions C1, C2 and C3, as well as double oxidations at C1/3, C2/3, or C3/4 of various sugars [14] and of certain aromatic glycosides [15]. The active sites of *Am*PDH and *Tm*P2O are also relatively similar, and their substrate preference is partly overlapping for the reductive half-reaction.

Modeling D-glucose in orientation for oxidation at C1, C2, C3 or C4 shows that the principal interactions are provided by the catalytic histidine pair and the backbone carbonyl oxygen of Tyr510 (Table S2; Fig. S1). In agreement with observed activity data [7], [28], the C2 and C3 oxidation modes appear very favorable (Fig. S1 *b, c*). *Tm*P2O has been shown to strongly favor the D-glucose C2 position for oxidation [4], [5]. In addition, our previous crystallographic studies on fluorinated glucose derivatives bound to *Tm*P2O provided a rationale for the discrimination of this enzyme against oxidation at the glucosyl C3 position [6]. With D-glucose as substrate, PDH was previously shown to display a slight preference for C3 over C2 [7], [16]. To evaluate the relative preference of *Am*PDH for D-glucose oxidation at C2 or C3, we performed reduction experiments using 3FG (C2 accessible) or 2FG (C3 accessible). Reduction of *Am*PDH by 3FG was considerably faster than by 2FG, showing that C2 is the preferred site of oxidation. Although, theoretically, the fluorine might influence the outcome of site attack, the pattern of regioselectivity for fluorinated and non-fluorinated glucose was consistent in the case of *Tm*P2O indicating that the fluorine does not significantly change the regioselectivity mechanism.

We may conclude that the observed substrate preferences of *Am*PDH can be satisfactorily rationalized by the crystal structure, and that steric clashes are less well tolerated in subsite C than in subsite B1. Subsite B1 is further out from the flavin pocket, and there is likely to be an increased ability for the protein to adjust and reposition loop regions to accommodate different substrates. In this context, it should be noted that only one structural conformer has been determined of *Am*PDH, and ligand-induced conformational changes may, as in the case of *Tm*P2O where regioselectivity is governed by active-site loop movements, recruit other protein groups to offer additional and differential interaction possibilities. However, the temperature factors are typically low for protein backbone atoms in site B1, and the electron density is well defined for all parts of the *Am*PDH active site, indicating that the conformer observed here is heavily dominating the conformational ensemble, at least in the present crystal form and in the absence of ligand. It should also be noted that extensive attempts have been made to capture ligand complexes of PDH using fluorinated glucoses, but without success so far.

### Oxygen Reactivity

Due to the potential generation of reactive oxygen species by molecular oxygen, biological redox processes involving oxygen are tightly controlled. The two-electron reduction of O_2_ by reduced singlet flavin is a spin-forbidden reaction, and to overcome this barrier, flavoenzymes have developed precisely tuned mechanisms to enable efficient catalysis of the one-electron O_2_ reduction reaction [31]. As pointed out recently [32], [33], any one single structural determinant in a flavoenzyme is unlikely to be solely and sufficiently responsible for oxygen reactivity. An identical side chain, equivalently positioned and oriented near the flavin, may be important for oxygen activation in one enzyme, but not in another, emphasizing the importance of context-dependent function.

In the case of flavoproteins, formation of a covalent flavin C(4a)-hydroperoxide adduct is one of the means for the enzymes to carry our monooxygenation (monooxygenase) or hydrogen peroxide elimination (oxidase) [34], [35]. This flavin species was considered to be limited to the monooxygenase reaction, but was recently reported also for the flavoprotein oxidase *Tm*P2O [36], [37]. A flavin C(4a)-adduct has also been detected in a crystalline state of the GMC enzyme choline oxidase [25], [38]. In the case of GOX, it has been suggested that the hydroperoxyflavin intermediate is bypassed in the oxygen activation mechanism, and instead, the superoxide anion is stabilized through direct interactions with protonated His516 [39], [40].

Suggested determinants of oxygen reactivity include thermodynamic and kinetic considerations, specifically the rate-limiting nature of the first thermodynamically unfavorable one-electron transfer to oxygen; as well as the possible presence of discrete oxygen channels and pockets for oxygen movement and capture [33], [39]. In the case of GOX, the positively charged His516 would offer stabilization to the superoxide, thus supporting sufficient rate enhancement of the oxygen reaction, while preventing the risk of accumulating toxic oxygen species [39]. Although these are clearly insightful and important observations, the structural origin of such effects is inherently difficult to assess and remain elusive.

Other studies emphasize the volume restriction of the flavoenzyme active site as a determinant of oxygen reactivity. One study concerns L-galactono-*γ*-lactone dehydrogenase (GALDH), a member of the *p*-cresol methylhydroxylase family of oxidoreductases [41]. GALDH displays some oxygen activity but performs poorly as an oxidase, however a single Ala→Gly replacement at position 113 was sufficient to convert the enzyme from a very poor oxidase to a highly competent oxidase [42]. This led the authors to suggest that Ala113 acts as a gatekeeper to prevent oxygen binding in wild-type GALDH, whereas the absence of a side chain creates enough space for oxygen to access the flavin ring. The authors provide an extensive analysis of amino-acid occurrences at this position in related enzymes, which implicated that the dehydrogenases feature Ala/Pro/Thr/Ser/Ile/Leu, whereas the oxidases have Gly/Ala/Pro [42].

A comparison of the position of the Ala105 Cβ atom in the enzyme alditol oxidase (PDB code 2VFS [43]) with that of Pro186 Cβ in cholesterol oxidase (type BCO2; PDB code 1I19 [44]) shows that they superimpose within 1 Å, a displacement that is correlated with the precise positioning of the isoalloxazine pyrimidine ring. Thus, the proline ring should be just as efficient in preventing oxygen binding as is the alanine side chain in a dehydrogenase. Moreover, cholesterol oxidase features a second proline (188) opposite to Pro186 which appears to restrict the space for a hypothetical oxygen molecule even further. These observations seriously challenge the assignment of this region as a general determinant of oxygen reactivity, and provide little support for the gatekeeper hypothesis, beyond possibly being relevant for GALDH. Nonetheless, the data mining carried out by Leferink and co-workers highlights the difficulties in generalizing structural determinants of oxygen activity even within a family of highly homologous structures. Recently, site-directed mutagenesis studies of aryl alcohol oxidase have shown the opposite effect when replacing a bulky sidechain (Phe501) with an alanine led to a ∼120-fold decrease in the oxygen activity, while the oxygen reactivity was increased ∼2-fold by the introduction of a larger Trp side chain [45].

Because of the obvious complexity of context dependency, great care has to be taken when analyzing structure, as well as biophysical and biochemical data. Preferably, enzymes that evolved structurally very similar active sites, but different oxygen reactivity, should be compared to simplify analysis of contextual interaction networks. The GMC family of oxidoreductases includes FAD-dependent oxidoreductases that share a common subunit fold, but represents a spectrum of electron-donor and electron-acceptor substrate specificities. Several members use sugars as electron donors, but display differences in electron-acceptor preference. The enzymes PDH, GOX and P2O represent an interesting triad where the active sites and electron-donor substrate specificities are sufficiently similar, and the oxygen reactivity significantly different, to allow a comparison: *i)* P2O displays oxygen reactivity and stabilizes a flavin C(4a) intermediate; *ii)* GOX displays oxygen reactivity but no flavin C(4a) adduct; and *iii)* PDH has no physiological oxygen reactivity but is able to physically form and stabilize a flavin C(4a) adduct.

As we show here, the oxygen reactivity of PDH is negligible, confirming that this reaction is not likely to be important physiologically and functionally. Notwithstandingly, it is interesting to note that *^i)^* PDH shows a red anionic flavin semiquinone, and that *^ii)^* the reduced enzyme forms a stable, long-lived flavin C(4a) adduct with an unidentified mono- or diatomic species under the conditions of the X-ray experiment. At this point, we make no claims that the flavin C(4a) adduct is a functionally relevant intermediate during the oxidative half-reaction of *Am*PDH, but rather, an artifact arising from attack by reactive oxygen species on the C(4a) position during irradiation of the protein crystal. The protein used for crystallization was in the reduced state, making such reactions possible. Presumably, the *Am*PDH preparation was reduced by sugars already inside the cell. The importance of the presence of the adduct species in *Am*PDH lies not in its tentative role, but in its well-defined structure, which provides insight regarding the structural details of this type of flavin intermediate in the active site of the closely related “oxygen-active” GMC members. Thus, this information can be extrapolated to the closely related active sites of GOX (PDB code 1CF3 [23]) and P2O (PDB code 1TT0 [46]) to evaluate possible structural requisites for oxygen reactivity, and activation (*i.e.*, flavin C(4a)-adduct formation and stabilization).

In *An*GOX, His516 has been pointed out as the critical residue for oxygen activation [40], [47], however, no spectral evidence has been obtained for a hydroperoxy-flavin intermediate. This histidine is conserved throughout the GMC family and it has been confirmed as the catalytic base in P2O [48]. If this histidine constitutes a general mechanism for oxygen activation, why would GMC members like PDH and CDH show no, or poor ability to activate oxygen? (see comparison in Table 2) The histidine may represent a necessary condition for O_2_ activation in the GMC-type flavoprotein oxidases (and possibly others), but is clearly not sufficient. Further comparison of related GMC flavoenzymes shows that neither of the determinants hitherto suggested in the literature is by itself a sufficient condition for oxygen reactivity (Table 2); *i.e.*, the principal histidine of the catalytic pair, protein flavinylation, formation of a flavin C(4a) adduct, or a stabilizing *si*-face N(5) factor.

In contrast to GOX, oxygen activation by *Tm*P2O has been confirmed to proceed *via* a flavin-C(4a)-hydroperoxide intermediate [36]. Indeed, we have shown previously that in order to support formation of a flavin-C(4a)-hydroperoxide intermediate in P2O, a fine-tuned environment around the flavin *re*-face below N(5) is critical for flavin C(4a)-adduct formation since replacing Thr169 by Ser, Ala or Gly effectively abolished C(4a)-hydroperoxy-flavin formation [49]. Similar results have also been observed in an FMN-dependent monooxygenase [50]. Using the reduced FAD specifically labeled at the flavin N(5) in P2O and solvent kinetic isotope effects, it was shown that the bond breakage of the flavin N(5)-H controls the overall process of H_2_O_2_ elimination [37]. The fine-tuned environment around the flavin N(5) is also important for enzyme flavinylation [51]. Without stabilizing flavin-C(4a)-hydroperoxide, the oxygen-activation mechanism of GOX has been suggested to involve a pre-organized superoxide anion-binding site [40], [47]. Based on the *Am*PDH adduct structure, one may conclude that there is definitely enough space in GOX to accommodate this intermediate, ruling out space limitation as a reason for lack of adduct formation in GOX.

An interesting observation is that *Tm*P2O, which is capable of both oxygen reactivity and flavin C(4a)-intermediate stabilization, features a distance of ∼4 Å between the side-chain nitrogen atoms of the catalytic pair, *i.e*., the N–N distance (Table 2). This distance is considerably shorter in the two sugar-oxidizing dehydrogenases that are unable to react with oxygen (∼3 Å in PDH and CDH), and longer in *An*GOX (∼5 Å), which reacts readily with oxygen but does not form a C(4a)-flavin intermediate. It should be noted that *Ag*CHO also displays an N–N distance of about 4 Å, but due to the uncertain relevance of the crystallographically observed adduct, we refrain from drawing any further conclusions regarding this enzyme.

Although it is too early to speculate on the general mechanistic significance of the N–N distance, undoubtedly, the intimate association of these two active-site side chains in the dehydrogenases leads to closer interaction not only with each other, but also with a hypothetical C(4a) adduct, however without generating steric hindrance or clashes. This is confirmed by the tight association of His512 Nε2 and His556 Nδ1 atoms with the C(4a)-oxygen species in *Am*PDH. At least for these GMC flavoenzymes, the N–N distance probably needs to be precisely controlled to offer the proper magnitude of intermediate stabilization needed for formation and decomposition of the adduct. A long N–N distance would offer no or little stabilization, whereas a very short distance would either abolish adduct formation altogether, or provide too strong stabilization. Clearly, electrostatic interactions [39], [52], and space requirements [41], [42] are likely to be of general importance for oxygen reactivity and the oxygen reaction mechanism. At least in the case of *Tm*P2O, which makes use of an oxygen-activation mechanism that takes place *via* the collapse of a caged radical pair to form the covalent flavin C(4a) intermediate that eventually leads to H_2_O_2_ elimination [37], the *si*-face partner (Thr169) constitutes a structural determinant for flavin C(4a) intermediate formation by stabilizing interactions at the flavin N(5)/O(4) locus [49]. A finely tuned interatomic distance between the two nitrogen atoms of the catalytic pair at the *re*-side of the N(5) locus may constitute an additional structural determinant. Drawing on its close structural and functional relationship to the sugar oxidases P2O and GOX, we anticipate that future mutagenesis and kinetic analyses of *Am*PDH, GOX and P2O will advance our understanding regarding oxygen reactivity and oxygen activation for this group of GMC oxidoreductases.

## Materials and Methods

### Expression and Purification

The *pdh1* gene from *A. meleagris* was heterologously expressed in *Pichia pastoris* under control of the inducible AOX promoter, and *Am*PDH was purified from the culture supernatant of a 60-L fed batch cultivation in principle as previously described [53]. In short, the purification protocol is based on hydrophobic interaction chromatography and anion exchange chromatography. A final step of size exclusion chromatography on a Superdex-75 column (GE Healthcare Life Sciences) equilibrated with 100 mM KH_2_PO_4_, pH 7.0 buffer containing 100 mM KCl was added to this published protocol to remove minor impurities. Fractions containing *Am*PDH activity were pooled, diafiltrated in 100 mM KH_2_PO_4_ buffer (pH 7.0) and concentrated.

### Re-oxidation of Reduced *Am*PDH

*Am*PDH is isolated mainly as the reduced enzyme. To generate the oxidized enzyme, *Am*PDH was mixed with 1 mM 2,6-dichlorophenol-indophenol (DCIP). The solution was centrifuged at 15,000 rpm for 10 min to remove precipitation of DCIP, and then passed through a gel-filtration column (Sephadex G25) pre-equilibrated with 100 mM NaH_2_PO_4_ (pH 7.5) to remove DCIP.

### Activity of Oxidized *Am*PDH with 2- and 3-Fluorinated Glucose as Electron Donor

To measure the reduction of *Am*PDH by fluorinated glucoses, either 20 µM 3FG or 20 µM 2FG, was added to 20 µM oxidized PDH in 100 mM NaH_2_PO_4_, pH 7.5 (prepared as above) in an anaerobic cuvette at 25°C. Absorption spectra of the reduction process were monitored at various intervals over a period of 1000 s.

### *Am*PDH Reactivity with Oxygen as Electron Acceptor

A solution of oxidized *Am*PDH (23 µM) was reduced with an equal amount of D-glucose to generate the reduced enzyme, and left at air-saturation ([O_2_] = 0.26 mM) for 15 hrs at 25°C. Absorption spectra of the reduced PDH reacting with 0.26 mM oxygen were recorded.

### Deglycosylation and Crystallization

A concentrated preparation of 90 mg *Am*PDH (60 mg/ml) was deglycosylated using a 30∶1 mass ratio of *Am*PDH and Jack bean α-mannosidase (Sigma-Aldrich®), specifically, 90 mg PDH and 3.1 mg (68 U) α-mannosidase. The reaction was performed in a solution containing 50 mM sodium acetate (pH 5.5) and 0.4 mM ZnCl_2_. The sample was incubated overnight at 37°C. Following digestion, the sample was applied onto a Superdex-75 column (GE Healthcare Life Sciences) in 50 mM HEPES (pH 6.5), 150 mM NaCl. Fractions containing *Am*PDH were pooled, diafiltrated in 50 mM HEPES buffer (pH 6.5) and concentrated. The purified protein was screened for crystallization under aerobic conditions using Crystal Screen (Hampton Research). Good quality crystals were obtained from formulation 42 with the addition of 2-methyl-2,4-pentanediol (MPD) [50 mM KH_2_PO_4_, 5% MPD, and 20% (w/v) polyethylene glycol 8,000], and microseeding. Prior to vitrification in liquid nitrogen, crystals were transferred to a cryo solution containing 50 mM KH_2_PO_4_, 5% MPD, 35% (v/w) polyethylene glycol 8,000. The crystals belong to space group *P*2_1_2_1_2_1_ with one molecule in the asymmetric unit and cell dimensions *a* = 52.315 Å, *b* = 75.170 Å, *c* = 140.212 Å. Intensity data were collected to 1.6 Å resolution at beamline *I*911-5 (*λ* = 0.90772 Å), MAX-lab, Lund, Sweden. Data were processed and scaled using the *XDS* package [54].

### Crystallographic Phasing and Refinement

The *Am*PDH structure was determined by molecular replacement using the fully automated *BALBES* pipeline [55] as implemented in *CCP4i* [56], [57], starting with diffraction and sequence data. Automated search model generation in *BALBES* tested five template models: 2JBV, 1CF3, 1GPE, 1JU2, 1KDG; with sequence identities to PDH of 26.1, 27.0, 25.3, 25.3, and 21.5%, respectively. The best Q value after all model calculations (0.5395) was obtained for 2JBV, which resulted in a suggested structure of 82% probability of being correct. After the integrated refinement with *REFMAC5* [58], the initial *R*/*R*_free_ values of 0.539/0.538 for this model decreased to 0.448/0.491. Following structure solution, the resulting partially refined model from *BALBES* was submitted for automated model building using the online ARP/wARP web service [59] available at the EMBL Hamburg website (http://www.embl-hamburg.de/ARP). Automatic model building resulted in a nearly complete model of 554 assigned residues (of 577) in eight chains and *R*/*R*_free_ values of 0.225 and 0.269, respectively. Refinement was performed using *REFMAC5* [56], [58], including anisotropic scaling, calculated hydrogen scattering from riding hydrogen atoms. Individual anisotropic B-factor refinement was performed for all atoms. Atomic displacement parameter refinement was performed using the translation, libration, screw-rotation (TLS) model. Eight TLS groups were defined, as determined by the TLS Motion Determination server, *TLSMD* [60]. Rebuilding and model manipulation were done using O [22] and Coot [61], guided by *σ*_A_-weighted 2*F_o_*–*F_c_* and *F_o_*–*F_c_* electron-density maps. Model validation was performed using *MolProbity* [62]. The 5000 K simulated annealing omit map shown in Fig. 7 was calculated using *PHENIX* [63]. Data collection and model refinement statistics are given in Table 1. All pictures showing structures were generated with PyMOL™ [64] (DeLano Scientific LLC, www.pymol.org). The atomic coordinates and structure factors (code 4H7U) for the *Am*PDH model have been deposited with the Protein Data Bank, Research Collaboratory for Structural Bioinformatics, Rutgers (http://www.rcsb.org).

### Modeling of Substrates in the *Am*PDH Active Site

To rationalize the observed patterns of substrate selectivity and regioselectivity for *Am*PDH [13], *in silico* modeling analyses were performed using substrates for which *^i)^* the relative activity is >50% of the activity for D-glucose, and *^ii)^* the site(s) of oxidation have been determined. The modeled substrates are shown in Fig. 1 and include: D-glucose, D-xylose, L-arabinose, D-galactose, methyl-α-D-glucose, methyl-β-D-glucose, cellobiose, maltose, and salicin [13], [14]. Modeling was performed by docking the individual substrates in the *Am*PDH active site using the program *O* [22], and identifying possible hydrogen bonds. The modeling was guided by the known binding modes for C2 and C3 oxidation of D-glucose determined for *Tm*P2O (PDB code 3PL8 [6] and 2IGO [3], respectively). Protein-sugar interactions were considered possible if appropriate hydrogen-bond donor and acceptor atoms are present within a distance of 3.3 Å. The coordinates for carbohydrate structures were retrieved using the HIC-Up database at Uppsala Software Factory (http://xray.bmc.uu.se/usf/), or in the case of salicin, generated from the published coordinates [65].

## Supporting Information

Figure S1 **Modeling of D-glucose in position for 1-, 2-, 3- and 4-oxidation.** The active site in *Am*PDH with D-glucose modeled in orientation for oxidation at (a) C1, (b) C2, (c) C3, and (d) C4. The protein is shown with beige carbon atoms, and the FAD cofactor and sugars in yellow and green, respectively.
(TIF)

Figure S2 **Modeling of D-xylose in position for 1-, 2-, 3- and 4-oxidation.** The active site in AmPDH with D-xylose modeled in orientation for oxidation at (a) C1, (b) C2, (c) C3, and (d) C4. The protein is shown with beige carbon atoms, and the FAD cofactor and sugar in yellow and green, respectively.
(TIF)

Figure S3 **Modeling of L-arabinose in position for 1-, 2-, 3- and 4-oxidation.** The active site in AmPDH with L-arabinose modeled in orientation for oxidation at (a) C1, (b) C2, (c) C3, and (d) C4. The protein is shown with beige carbon atoms, and the FAD cofactor and sugar in yellow and green, respectively.
(TIF)

Figure S4 **Modeling of D-galactose in position for 1-, 2-, 3- and 4-oxidation.** The active site in AmPDH with D-galactose modeled in orientation for oxidation at (a) C1, (b) C2, (c) C3, and (d) C4. The protein is shown with beige carbon atoms, and the FAD cofactor and sugar in yellow and green, respectively.
(TIF)

Figure S5 **Modeling of methyl-α-D-glucose in position for 1-, 2-, 3- and 4-oxidation.** The active site in AmPDH with methyl-α-D-glucose modeled in orientation for oxidation at (a) C1, (b) C2, (c) C3, and (d) C4. The protein is shown with beige carbon atoms, and the FAD cofactor and sugar in yellow and green, respectively.
(TIF)

Figure S6 **Modeling of methyl-β-D-glucose in position for 1-, 2-, 3- and 4-oxidation.** The active site in AmPDH with methyl-β-D-glucose modeled in orientation for oxidation at (a) C1, (b) C2, (c) C3, and (d) C4. The protein is shown with beige carbon atoms, and the FAD cofactor and sugar in yellow and green, respectively.
(TIF)

Figure S7 **Modeling of cellobiose in position for 1-, 2-, 3-, 2′-, 3′- and 4′-oxidation.** The active site in AmPDH with cellobiose modeled in orientation for oxidation at (a) C1, (b) C2, (c) C3, (d) C2′, (e) C3′, and (f) C4′. The protein is shown with beige carbon atoms, and the FAD cofactor and sugar in yellow and green, respectively.
(TIF)

Figure S8 **Modeling of maltose in position for 1-, 2-, 3-, 2′-, 3′- and 4′-oxidation.** The active site in AmPDH with maltose modeled in orientation for oxidation at (a) C1, (b) C2, (c) C3, (d) C2′, (e) C3′, and (f) C4′. The protein is shown with beige carbon atoms, and the FAD cofactor and sugar in yellow and green, respectively.
(TIF)

Figure S9 **Modeling of salicin in position for 1-, 2-, 3- and 4-oxidation.** The active site in AmPDH with salicin modeled in orientation for oxidation at (a) C1, (b) C2, (c) C3, and (d) C4. The protein is shown with beige carbon atoms, and the FAD cofactor and sugar in yellow and green, respectively.
(TIF)

Table S1 **Geometry of flavin C(4a) O-adducts in known crystal structures.**
(DOCX)

Table S2 **Computational modeling of monosaccharides in the** ***Am*** **PDH active site.**
(DOCX)

Table S3 **Computational modeling of disaccharides in the** ***Am*** **PDH active site.**
(DOCX)
