# Supplementary figures and images for: The 1.6 Å Crystal Structure of Pyranose Dehydrogenase from Agaricus meleagris Rationalizes Substrate Specificity and Reveals a Flavin Intermediate
Source: PLoS One. 2013 Jan 9;8(1):e53567. doi: 10.1371/journal.pone.0053567 (PMC3541233; doi:10.1371/journal.pone.0053567)

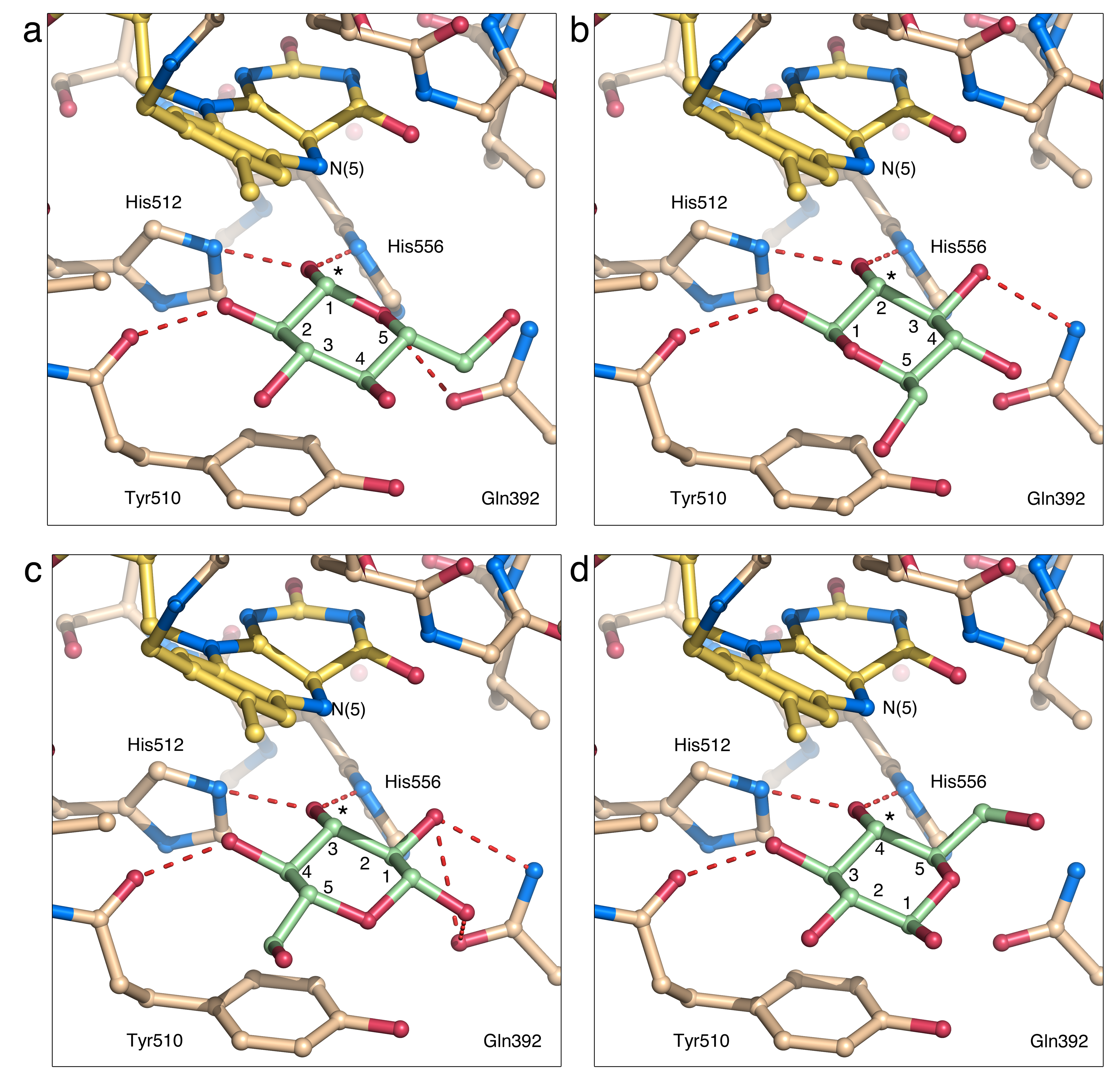

Supplement: Figure S1 — Modeling of D-glucose in position for 1-, 2-, 3- and 4-oxidation. The active site in AmPDH with D-glucose modeled in orientation for oxidation at (a) C1, (b) C2, (c) C3, and (d) C4. The protein is shown with beige carbon atoms, and the FAD cofactor and sugars in yellow and green, respectively. (TIF) [file pone.0053567.s001.tif]

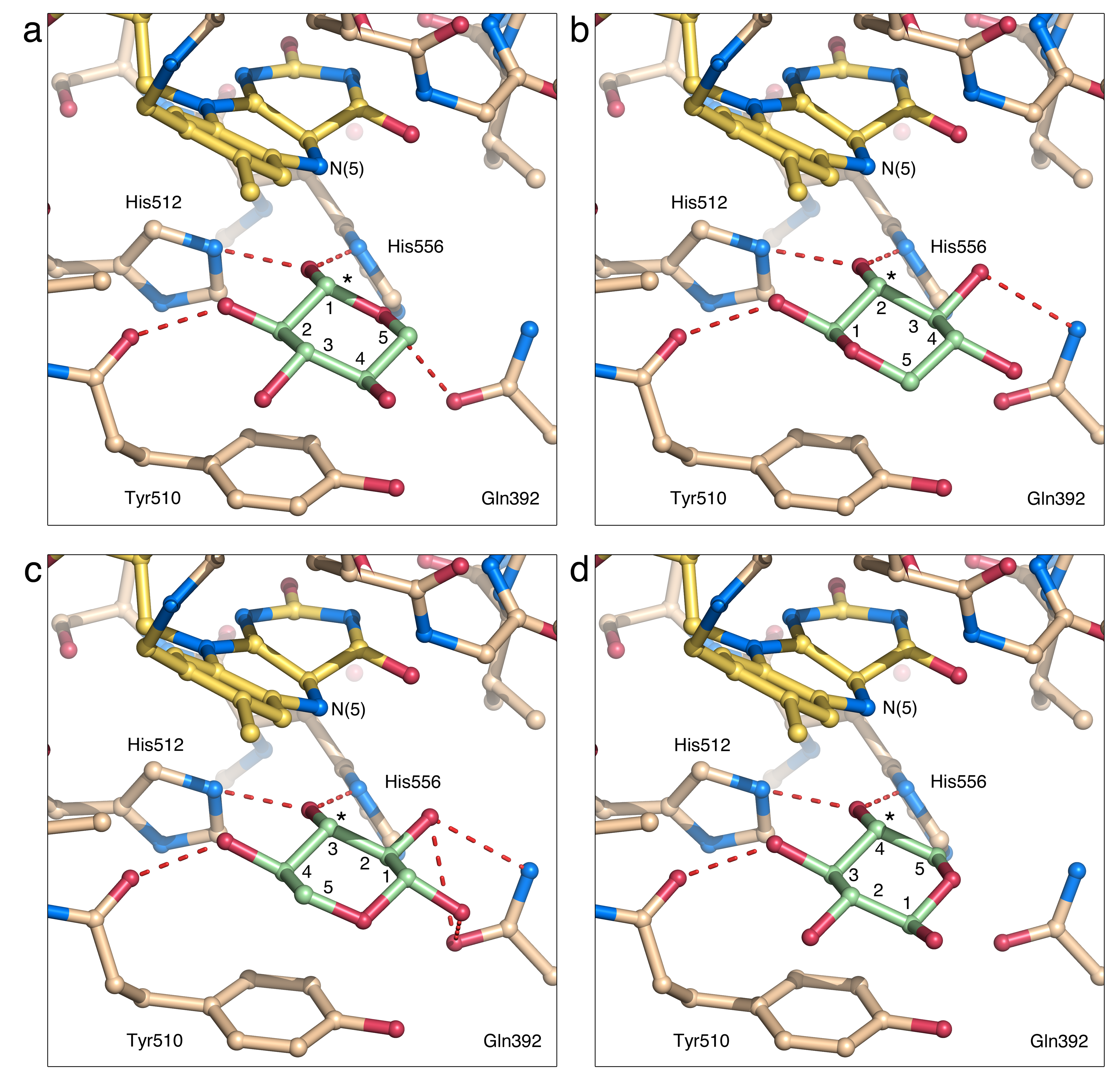

Supplement: Figure S2 — Modeling of D-xylose in position for 1-, 2-, 3- and 4-oxidation. The active site in AmPDH with D-xylose modeled in orientation for oxidation at (a) C1, (b) C2, (c) C3, and (d) C4. The protein is shown with beige carbon atoms, and the FAD cofactor and sugar in yellow and green, respectively. (TIF) [file pone.0053567.s002.tif]

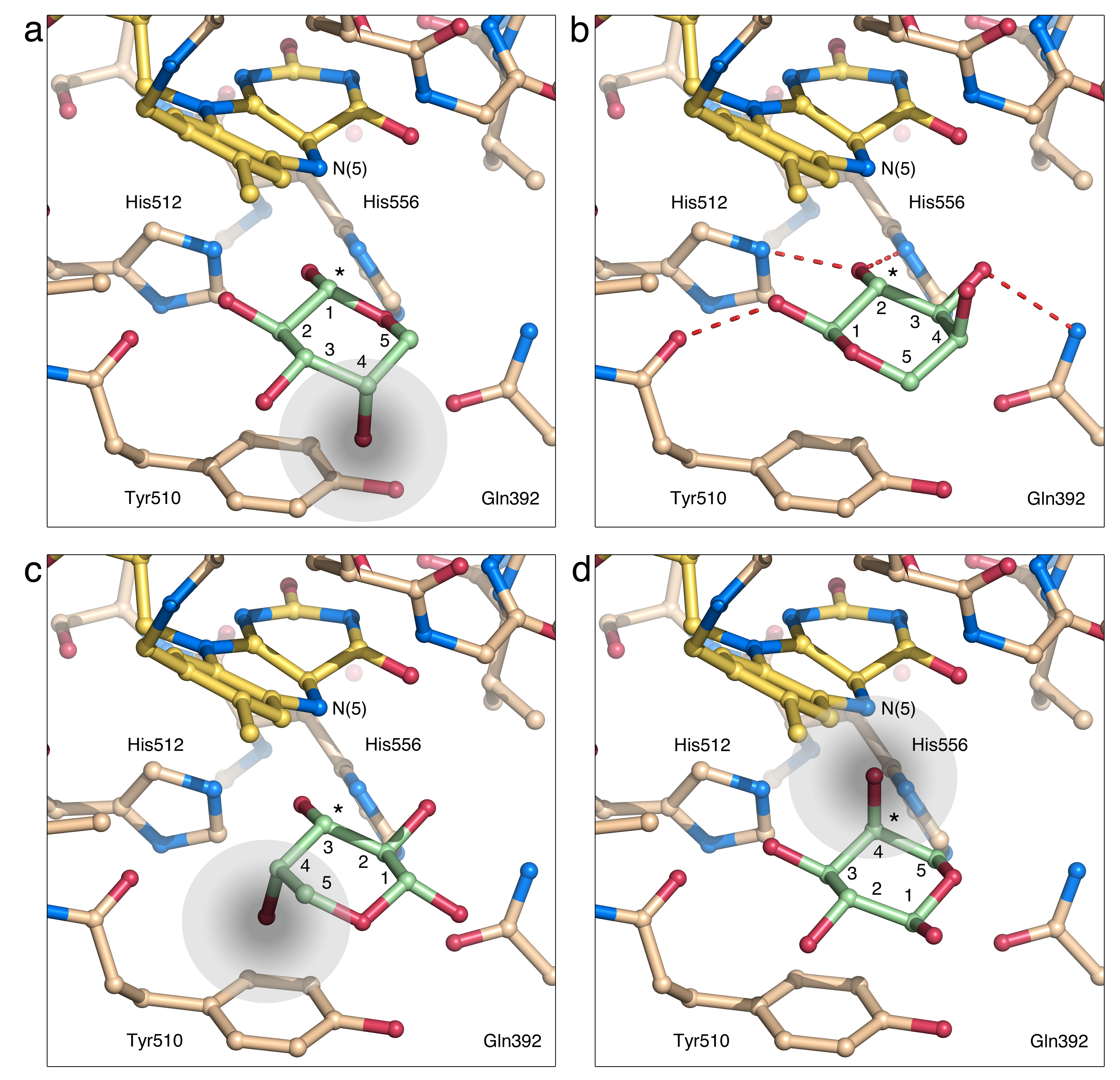

Supplement: Figure S3 — Modeling of L-arabinose in position for 1-, 2-, 3- and 4-oxidation. The active site in AmPDH with L-arabinose modeled in orientation for oxidation at (a) C1, (b) C2, (c) C3, and (d) C4. The protein is shown with beige carbon atoms, and the FAD cofactor and sugar in yellow and green, respectively. (TIF) [file pone.0053567.s003.tif]

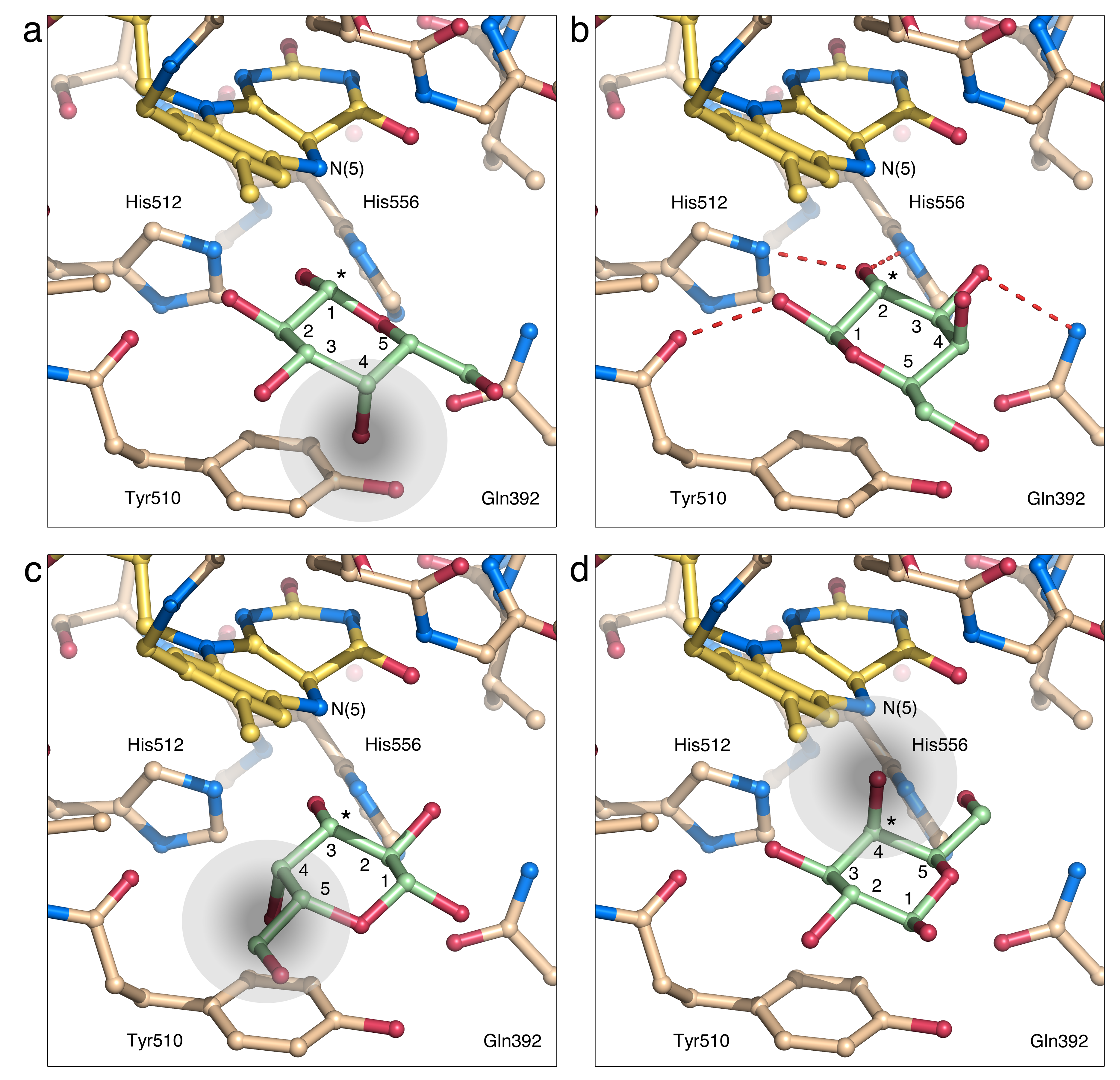

Supplement: Figure S4 — Modeling of D-galactose in position for 1-, 2-, 3- and 4-oxidation. The active site in AmPDH with D-galactose modeled in orientation for oxidation at (a) C1, (b) C2, (c) C3, and (d) C4. The protein is shown with beige carbon atoms, and the FAD cofactor and sugar in yellow and green, respectively. (TIF) [file pone.0053567.s004.tif]

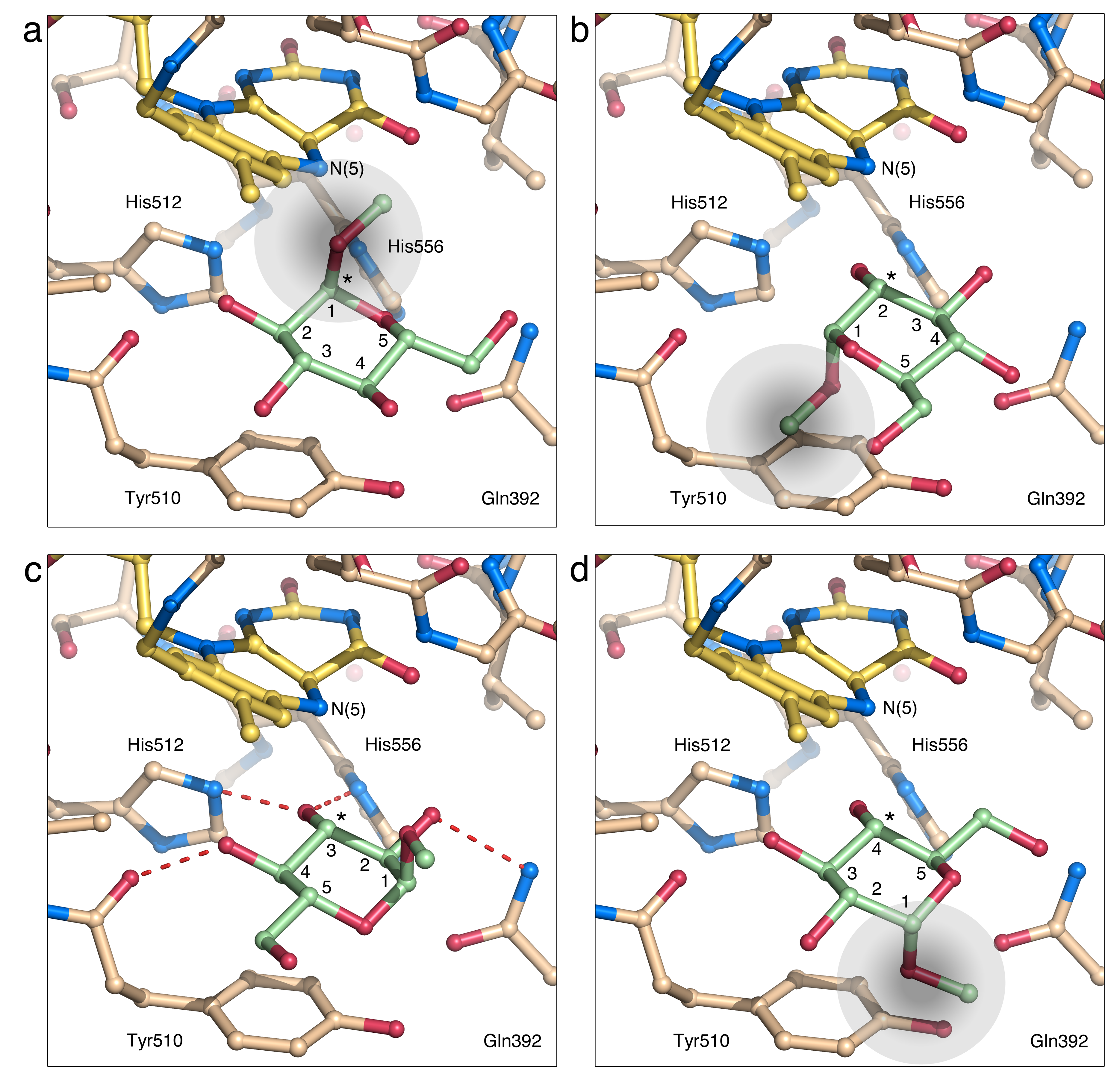

Supplement: Figure S5 — Modeling of methyl-α-D-glucose in position for 1-, 2-, 3- and 4-oxidation. The active site in AmPDH with methyl-α-D-glucose modeled in orientation for oxidation at (a) C1, (b) C2, (c) C3, and (d) C4. The protein is shown with beige carbon atoms, and the FAD cofactor and sugar in yellow and green, respectively. (TIF) [file pone.0053567.s005.tif]

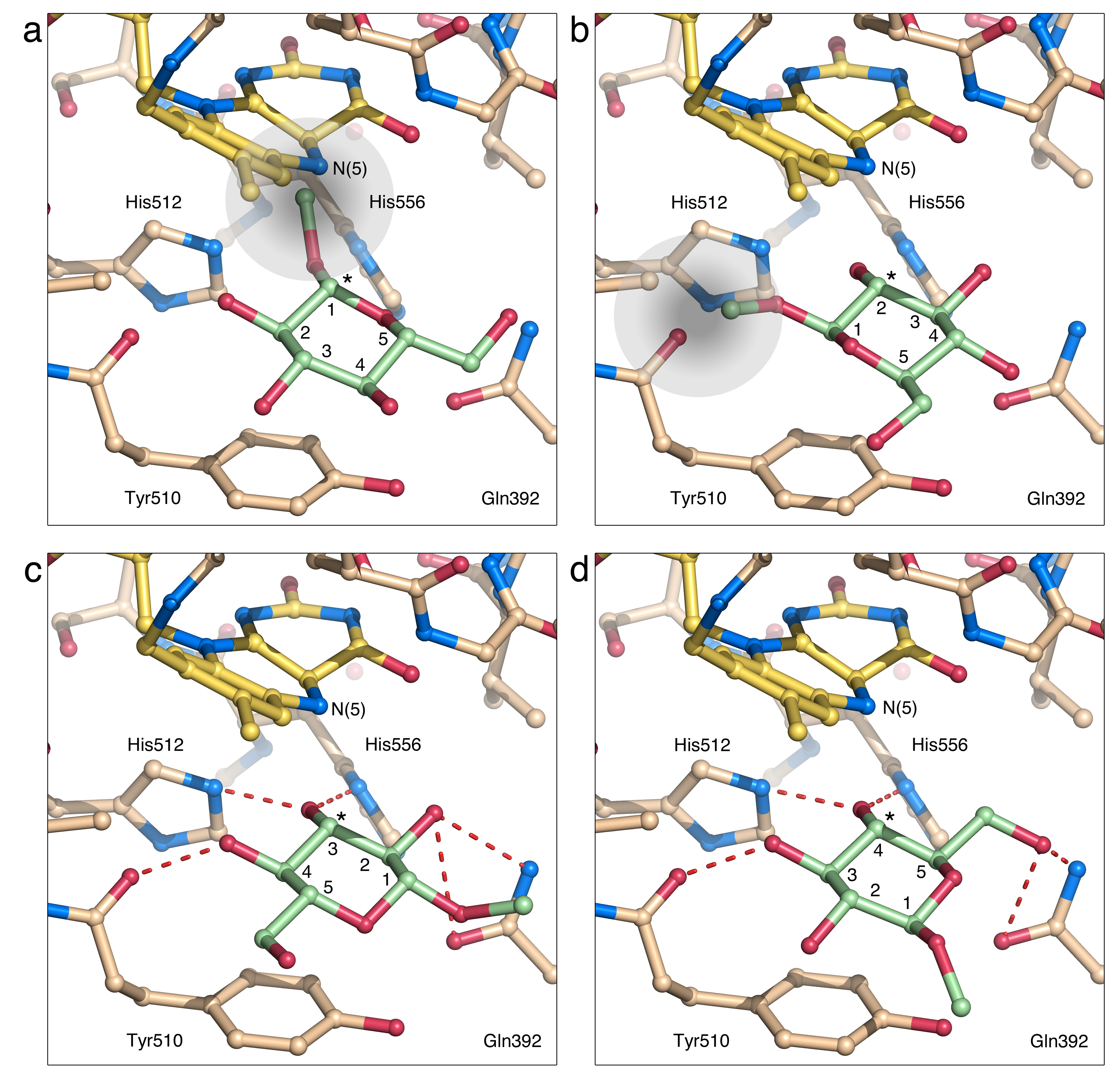

Supplement: Figure S6 — Modeling of methyl-β-D-glucose in position for 1-, 2-, 3- and 4-oxidation. The active site in AmPDH with methyl-β-D-glucose modeled in orientation for oxidation at (a) C1, (b) C2, (c) C3, and (d) C4. The protein is shown with beige carbon atoms, and the FAD cofactor and sugar in yellow and green, respectively. (TIF) [file pone.0053567.s006.tif]

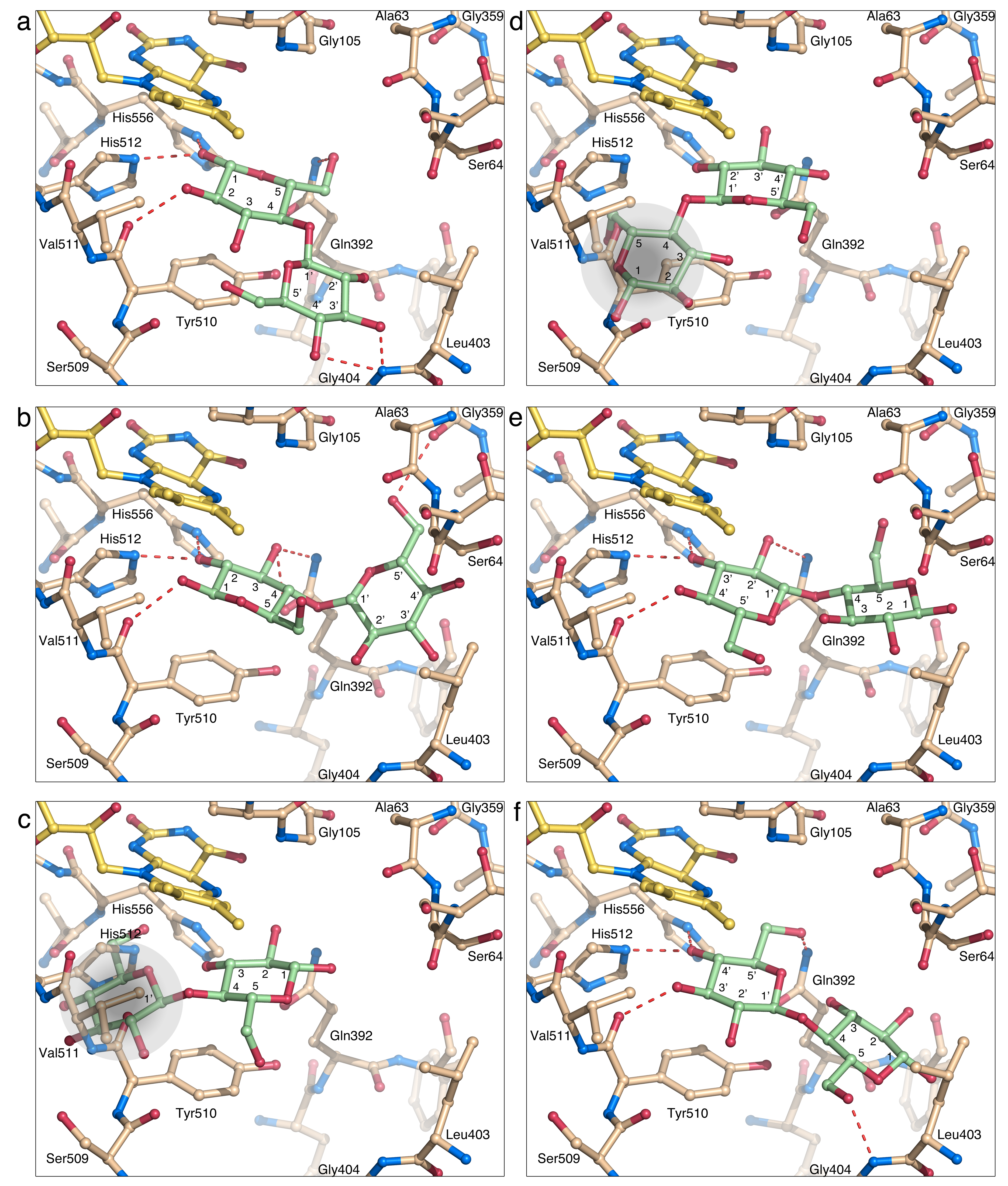

Supplement: Figure S7 — Modeling of cellobiose in position for 1-, 2-, 3-, 2′-, 3′- and 4′-oxidation. The active site in AmPDH with cellobiose modeled in orientation for oxidation at (a) C1, (b) C2, (c) C3, (d) C2′, (e) C3′, and (f) C4′. The protein is shown with beige carbon atoms, and the FAD cofactor and sugar in yellow and green, respectively. (TIF) [file pone.0053567.s007.tif]

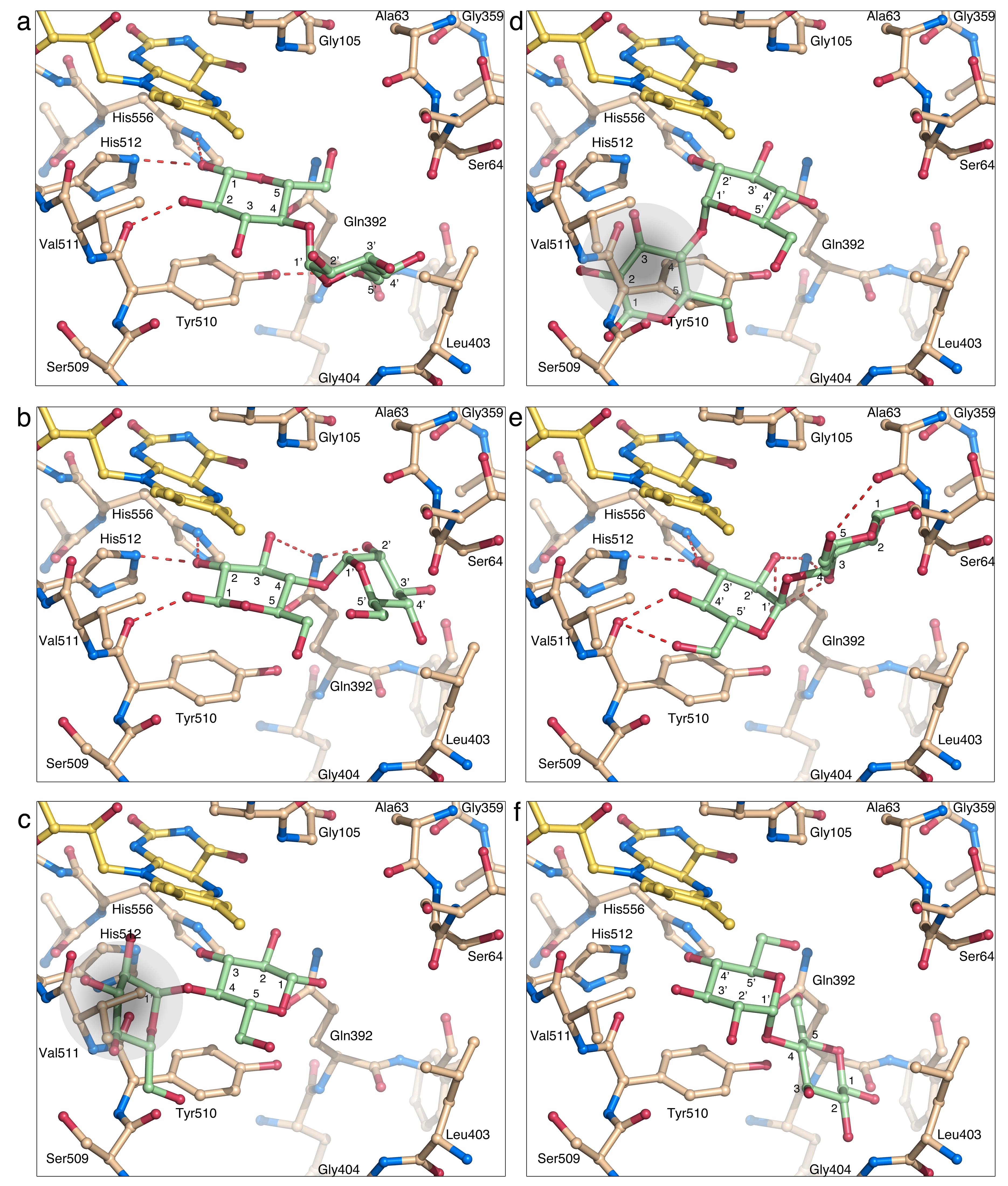

Supplement: Figure S8 — Modeling of maltose in position for 1-, 2-, 3-, 2′-, 3′- and 4′-oxidation. The active site in AmPDH with maltose modeled in orientation for oxidation at (a) C1, (b) C2, (c) C3, (d) C2′, (e) C3′, and (f) C4′. The protein is shown with beige carbon atoms, and the FAD cofactor and sugar in yellow and green, respectively. (TIF) [file pone.0053567.s008.tif]

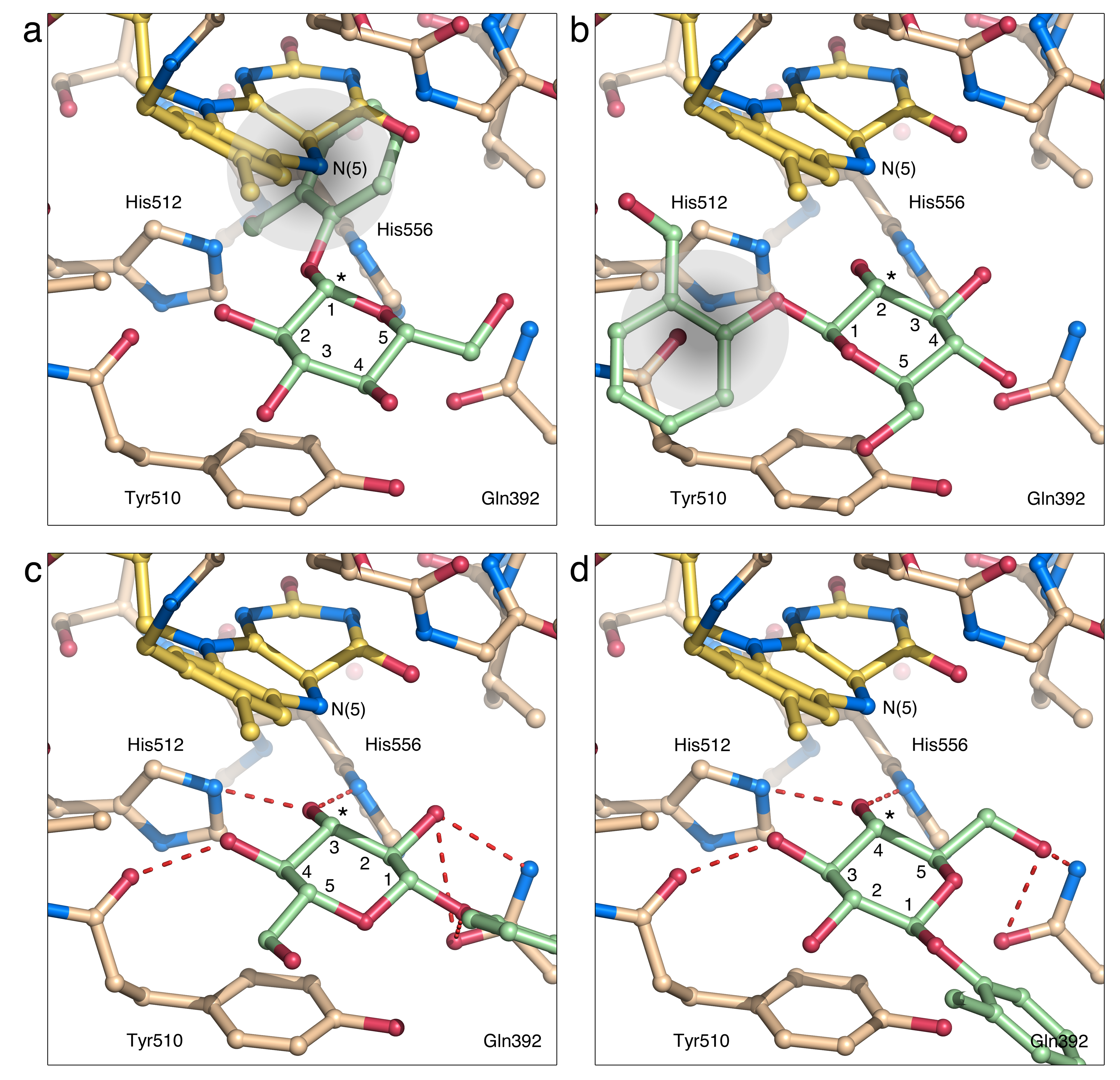

Supplement: Figure S9 — Modeling of salicin in position for 1-, 2-, 3- and 4-oxidation. The active site in AmPDH with salicin modeled in orientation for oxidation at (a) C1, (b) C2, (c) C3, and (d) C4. The protein is shown with beige carbon atoms, and the FAD cofactor and sugar in yellow and green, respectively. (TIF) [file pone.0053567.s009.tif]
