# Supplementary material for: The 1.6 Å Crystal Structure of Pyranose Dehydrogenase from Agaricus meleagris Rationalizes Substrate Specificity and Reveals a Flavin Intermediate
Source: PLoS One. 2013 Jan 9;8(1):e53567. doi: 10.1371/journal.pone.0053567 (PMC3541233; doi:10.1371/journal.pone.0053567)
Supplement: Table S1 — Geometry of flavin C(4a) O-adducts in known crystal structures. (DOCX) [file pone.0053567.s010.docx]

**Table S1. Geometry of flavin C(4a) O-adducts in known crystal structures**

|  | 1AOH^a^ | 1BOH^a^ | *Am*PDH; (this work) | DFT; C(4a)–OH / –OOH^b^ | CHO; C(4a)–OH / –OOH^b^ |
| --- | --- | --- | --- | --- | --- |
| Bond lengths (Å) | | | | | |
| C(4a)–O | 1.41 | 1.43 | 1.38 | 1.43 / 1.42 | 1.43 / 1.42 |
| C(4a)–C(10a) | 1.52 | 1.52 | 1.47 | 1.52 / 1.51 | 1.47 / 1.46 |
| C(4a)–C(4) | 1.52 | 1.55 | 1.44 | 1.56 / 1.59 | 1.59 / 1.59 |
| C(4a)–N(5) | 1.45 | 1.44 | 1.49 | 1.43 / 1.43 | 1.47 / 1.48 |
| Bond angles (°) | | | | | |
| C(4)–C(4a)–O(4a) | 105.9 | 103.2 | 103.9 | 106.1 / 100.7 | 123.5 / 119.0 |
| N(5)–C(4a)–O(4a) | 110.4 | 113.1 | 104.8 | 109.2 / 110.7 | 100.7 / 105.4 |
| C(10a)–C(4a)–O(4a) | 104.5 | 108.9 | 102.9 | 110.3 / 110.9 | 107.4 / 110.9 |
| C(4)–C(4a)–N(5) | 111.9 | 110.6 | 114.8 | 111.8 / 113.3 | 120.8 / 122.9 |
| C(4)–C(4a)–C(10a) | 112.9 | 106.7 | 109.6 | 104.7 / 106.0 | 94.6 / 85.5 |
| C(10a)–C(4a)–N(5) | 110.8 | 113.6 | 118.8 | 114.3 / 114.5 | 110.7 / 111.3 |
| Dihedral angles (°) | | | | | |
| O(4a)–C(4a)–N(5)–C(5a) | 60.9 | 94.2 | –108.7 | –125.1 / –134.0 | –147.5 / –156.0 |
| O(4a)–C(4a)–C(4)–N(3) | –101.6 | –70.6 | 68.7 | 70.3 / 69.3 | 44.1 / 43.5 |
| N(3)–C(4)–C(4a)–N(5) | 138.0 | 168.1 | –177.3 | –170.8 / –172.5 | 175.5 / 179.5 |
| C(4)–C(4a)–N(5)–C(5a) | 178.7 | –150.5 | 137.9 | 117.8 / 113.8 | 71.8 / 63.1 |
| C(4a)–N(5)–C(5a)–C(6) | –151.6 | –158.9 | –167.0 | –179.1 / –172.2 | –168.9 / –163.0 |
| N(5)–C(5a)–C(6)–C(7) | 175.6 | –176.6 | 166.1 | 168.1 / 169.4 | –177.9 / –178.1 |
| C(2)–N(1)–C(10a)–N(10) | –179.7 | –169.2 | 172.5 | 162.1 / 167.3 | 94.9 / 107.6 |
| N(1)–C(10a)–N(10)-C(9a) | 167.1 | –171.9 | –174.9 | –167.0 / –159.6 | –173.1 / 175.7 |
| C(10a)–N(10)–C(9a)–C(9) | 164.4 | 168.3 | 156.0 | 161.0 / 155.0 | –164.4 / –176.0 |
| N(10)-C(9a)–C(9)–C(8) | 173.2 | 178.1 | –176.4 | –171.9 / –173.8 | –179.1 / –177.3 |
| N(3)–C(4)–C(4a)–C(10a) | 12.2 | 44.1 | –40.7 | –46.5 / –46.2 | –67.3 / –68.0 |
| N(1)–C(10a)–C(4a)–C(4) | –3.5 | –45.1 | 45.2 | 51.2 / 47.7 | 59.7 / 73.5 |
| C(4)–C(4a)–C(10a)-N(10) | 174.5 | 139.7 | –152.0 | –128.9 / –132.1 | –72.9 / –86.0 |
| N(1)–C(10a)–C(4a)–N(5) | –129.8 | –167.2 | 179.9 | 173.9 / 173.2 | –175.6 / –162.9 |
| Improper dihedral angles (°) | | | | | |
| N(1)–N(10)–N(5)–C(6) | –173.6 | 169.9 | 167.3 | not reported | –177.3 |
| C(9)–N(10)–N(5)–C(4) | –155.6 | 173.5 | –150.6 | not reported | –131.3 |
| C(10a)–N(10)–N(5)–C(5a) | 172.0 | 166.8 | 168.4 | not reported | –176.7 |
| C(4a)–N(5)–N(10)–C(9a) | –139.5 | –158.2 | –175.8 | not reported | –165.9 |

^a^ Menova P, Eugner V, Cejka J, Dvorakova H, Sanda M, et al. (2011) Synthesis and structural studies of flavin and alloxazine adducts with O-nucleophiles. J Mol Struct 1004: 178–187.

^b^ Orville AM, Lountos GT, Finnegan S, Gadda G (2009) Crystallographic, spectroscopic, and computational analysis of a flavin C4a-oxygen adduct in choline oxidase. Biochemistry 48: 720–728.
