# Supplementary material for: The 1.6 Å Crystal Structure of Pyranose Dehydrogenase from Agaricus meleagris Rationalizes Substrate Specificity and Reveals a Flavin Intermediate
Source: PLoS One. 2013 Jan 9;8(1):e53567. doi: 10.1371/journal.pone.0053567 (PMC3541233; doi:10.1371/journal.pone.0053567)
Supplement: Table S2 — Computational modeling of monosaccharides in the Am PDH active site. (DOCX) [file pone.0053567.s011.docx]

**Table S2. Computational modeling of monosaccharides in the *Am*PDH active site**

| Substrate | RA (%)^a^ | Preferred oxidation sites | Site | Possible sugar-protein interactions in subsite C^b^ |
| --- | --- | --- | --- | --- |
| D-xylose | 113 | 2 + 3 + 2,3^c^ | C1 | O1–His556 Nδ1; O1–His512 Nε2; O2–Tyr510 O; O5–Gln392 Oε1 |
|  |  |  | C2 | O2–His556 Nδ1; O2–His512 Nε2; O1–Tyr510 O; O3-Gln392 Nε2 |
|  |  |  | C3 | O3–His556 Nδ1; O3–His512 Nε2; O4–Tyr510 O; O2–Gln392 Oε1; O2-Gln392 Nε2; O1–Gln392 Oε1 |
|  |  |  | C4 | O4–His556 Nδ1; O4–His512 Nε2; O3–Tyr510 O; O6–Gln392 Oε1; O6-Gln392 Nε2 |
| Methyl-α-D-Glc*p* | 107 | 3^d^ | C1 | not possible, severe clash between 1-methyl group and flavin N(5) locus |
|  |  |  | C2 | not possible, severe clash between 1-methyl group and Tyr510 ring |
|  |  |  | C3 | O3–His556 Nδ1; O3–His512 Nε2; O4–Tyr510 O; O2–Gln392 Oε1; O2-Gln392 Nε2 |
|  |  |  | C4 | not possible, severe clash between 1-methyl group and Tyr510 ring |
| D-glucose | 100 | 2 + 3 + 2,3^e,f^ | C1 | O1–His556 Nδ1; O1–His512 Nε2; O2–Tyr510 O; O6–Gln392 Oε1; O6-Gln392 Nε2; O5–Gln392 Oε1 |
|  |  |  | C2 | O2–His556 Nδ1; O2–His512 Nε2; O1–Tyr510 O; O3-Gln392 Nε2 |
|  |  |  | C3 | O3–His556 Nδ1; O3–His512 Nε2; O4–Tyr510 O; O2–Gln392 Oε1; O2-Gln392 Nε2; O1–Gln392 Oε1 |
|  |  |  | C4 | O4–His556 Nδ1; O4–His512 Nε2; O3–Tyr510 O; O6–Gln392 Oε1; O6-Gln392 Nε2 |
| L-arabinose | 100 | 2^g^ | C1 | not possible, axial O4 clashes with Tyr510 ring |
|  |  |  | C2 | O2–His556 Nδ1; O2–His512 Nε2; O1–Tyr510 O; O3-Gln392 Nε2 |
|  |  |  | C3 | not possible, axial O4 clashes with Tyr510 ring |
|  |  |  | C4 | not possible, axial O4 clashes with flavin ring |
| D-galactose | 99 | 2^f^ | C1 | not possible, axial O4 clashes with Tyr510 ring |
|  |  |  | C2 | O2–His556 Nδ1; O2–His512 Nε2; O1–Tyr510 O; O3-Gln392 Nε2 |
|  |  |  | C3 | not possible, axial O4 clashes with Tyr510 ring |
|  |  |  | C4 | not possible, axial O4 clashes with flavin ring |
| Salicin | 78 | 3 + 3,4^h^ | C1 | not possible, benzylic ring of salicin clashes with flavin ring |
|  |  |  | C2 | not possible, benzylic alcohol ring of salicin clashes with Tyr510 backbone |
|  |  |  | C3 | O3–His556 Nδ1; O3–His512 Nε2; O4–Tyr510 O; O2–Gln392 Oε1; O2-Gln392 Nε2; O1–Gln392 Oε1 |
|  |  |  | C4 | O4–His556 Nδ1; O4–His512 Nε2; O3–Tyr510 O; O6–Gln392 Oε1; O6-Gln392 Nε2 |
| Methyl-β-D-Glc*p* | 58 | 3^d^ | C1 | not possible, 1-methyl group clashes with flavin ring |
|  |  |  | C2 | not possible, 1-methyl group clashes with Tyr510 O and Val511 Cγ2 |
|  |  |  | C3 | O3–His556 Nδ1; O3–His512 Nε2; O4–Tyr510 O; O2–Gln392 Oε1; O2-Gln392 Nε2 |
|  |  |  | C4 | O4–His556 Nδ1; O4–His512 Nε2; O3–Tyr510 O; O6–Gln392 Oε1; O6-Gln392 Nε2 |

^a^ RA, relative activities exceeding 50% of the activity for D-glucose as taken from [Sedmera P, Halada P, Kubatova E, Haltrich D, Prikrylova V, et al. (2006) New biotransformations of some reducing sugars to the corresponding (di)dehydro(glycosyl) aldoses or aldonic acids using fungal pyranose dehydrogenase. J Mol Catal B 41: 32–42].

^b^ Blue fields, structure supports and explains published activity data; red fields, binding not possible; green fields, the structure supports binding, but activity data have not been reported; n.a. not applicable; n.d. not detected; possible interactions defined as < 3.3 Å between appropriate hydrogen-bond donor and acceptor.

^c^ Volc J, Sedmera P, Halada P, Prikrylova V, Haltrich D (2000) Double oxidation of D-xylose to D-glycero-pentos-2,3-diulose (2,3-diketo-D-xylose) by pyranose dehydrogenase from the mushroom *Agaricus bisporus*. Carbohydr Res 329: 219–225.

^d^ Volc J, Sedmera P, Halada P, Daniel G, Prikrylova V, et al. (2002) C-3 oxidation of non-reducing sugars by a fungal pyranose dehydrogenase: spectral characterization. J Mol Catal B Enzym 17: 91–100.

^e^ Volc J, Kubatova E, Daniel G, Sedmera P, Haltrich D (2001) Screening of basidiomycete fungi for the quinone-dependent sugar C-2/C-3 oxidoreductase, pyranose dehydrogenase, and properties of the enzyme from *Macrolepiota rhacodes*. Arch Microbiol 176: 178–186.

^f^ Volc J, Sedmera P, Halada P, Prikrylov V, Daniel G (1998) C-2 and C-3 oxidation of D-Glc, and C-2 oxidation of D-Gal by pyranose dehydrogenase from *Agaricus bisporus*. Carbohydr Res 310: 151–156.

^g^ Sedmera P, Halada P, Kubatova E, Haltrich D, Prikrylova V, et al. (2006) New biotransformations of some reducing sugars to the corresponding (di)dehydro(glycosyl) aldoses or aldonic acids using fungal pyranose dehydrogenase. J Mol Catal B 41: 32–42.

^h^ Sedmera P, Halada P, Peterbauer C, Volc J (2004) A new enzyme catalysis: 3,4-dioxidation of some aryl β-D-glycopyranosides by fungal pyranose dehydrogenase. Tetrahedron Lett 45: 8677–8680.
