# Supplementary material for: The 1.6 Å Crystal Structure of Pyranose Dehydrogenase from Agaricus meleagris Rationalizes Substrate Specificity and Reveals a Flavin Intermediate
Source: PLoS One. 2013 Jan 9;8(1):e53567. doi: 10.1371/journal.pone.0053567 (PMC3541233; doi:10.1371/journal.pone.0053567)
Supplement: Table S3 — Computational modeling of disaccharides in the Am PDH active site. (DOCX) [file pone.0053567.s012.docx]

**Table S3. Computational modeling of disaccharides in the *Am*PDH active site**

| Substrate | RA (%)^a^ | Preferred oxidation sites | Oxidation site | Possible sugar-protein interactions in subsite C^b^ | Possible sugar-protein interactions in subsite B1 |
| --- | --- | --- | --- | --- | --- |
| Cellobiose | 70 | 1 + 2 + 3′ + 1,3′ + 2,3′^c^ | C1 | O1–His556 Nδ1; O1–His512 Nε2; O2–Tyr510 O; O6-Gln392 Nε2 | O3′–Gly404 N; O4′–Gly404 N |
|  |  |  | C2 | O2–His556 Nδ1; O2–His512 Nε2; O1– Tyr510 O; O3–Gln392 Oε1; O3-Gln392 Nε2 | O6′–Gly359 O |
|  |  |  | C3 | not possible, severe clashes | n.a. |
|  |  |  | C2′ | not possible, severe clashes | n.a. |
|  |  |  | C3′ | O3′–His556 Nδ1; O3′–His512 Nε2; O4′–Tyr510 O; O2′-Gln392 Nε2 | no interactions |
|  |  |  | C4′ | O4′–His556 Nδ1; O4′–His512 Nε2; O3′–Tyr510 O; O6′–Gln392 Nε2 | O6–Gly404 N |
| Maltose | 61 | 1 + 2 + 3′ + 1,3′ + 2,3′^c^ | C1 | O1–His556 Nδ1; O1–His512 Nε2; O2–Tyr510 O | O6′–Tyr510 Oη |
|  |  |  | C2 | O2–His556 Nδ1; O2–His512 Nε2; O1–Tyr510 O; O3-Gln392 Nε2 | O2′–Gln392 Nε2 |
|  |  |  | C3 | not possible, severe clashes | n.a. |
|  |  |  | C2′ | not possible, severe clashes | n.a. |
|  |  |  | C3′ | O3′–His556 Nδ1; O3′–His512 Nε2; O4′–Tyr510 O; O6′– Tyr510 O; O2′–Gln392 Oε1; O2′-Gln392 Nε2 | O3–Gln392 Oε1; O3-Gln392 Nε2; O6–Ala63 O |
|  |  |  | C4′ | not possible, severe clashes | n.a. |
| Salicin | 78 | 3 + 3,4^d^ | C1 | not possible, severe clashes | no interactions |
|  |  |  | C2 | not possible, severe clashes | no interactions |
|  |  |  | C3 | O3–His556 Nδ1; O3–His512 Nε2; O4–Tyr510 O; O2–Gln392 Oε1; O2-Gln392 Nε2; O1–Gln392 Oε1 | benzylic ring in site B1 |
|  |  |  | C4 | O4–His556 Nδ1; O4–His512 Nε2; O3–Tyr510 O; O6–Gln392 Oε1; O6-Gln392 Nε2 | benzylic ring in site B1 |

^a^ RA, relative activities exceeding 50% of the activity for D-glucose as taken from [Sedmera P, Halada P, Kubatova E, Haltrich D, Prikrylova V, et al. (2006) New biotransformations of some reducing sugars to the corresponding (di)dehydro(glycosyl) aldoses or aldonic acids using fungal pyranose dehydrogenase. J Mol Catal B 41: 32–42].

^b^ Blue fields, structure supports and explains published activity data; red fields, binding not possible; green fields, the structure supports binding, but activity data have not been reported; n.a. not applicable; n.d. not detected; possible interactions defined as < 3.3 Å between appropriate hydrogen-bond donor and acceptor

^c^ Sedmera P, Halada P, Kubatova E, Haltrich D, Prikrylova V, et al. (2006) New biotransformations of some reducing sugars to the corresponding (di)dehydro(glycosyl) aldoses or aldonic acids using fungal pyranose dehydrogenase. J Mol Catal B 41: 32–42.

^d^ Volc J, Sedmera P, Halada P, Daniel G, Prikrylova V, et al. (2002) C-3 oxidation of non-reducing sugars by a fungal pyranose dehydrogenase: spectral characterization. J Mol Catal B Enzym 17: 91–100.
